# Green micellar UPLC and complementary eco-friendly spectroscopic techniques for simultaneous analysis of anti-COVID drugs: a comprehensive evaluation of greenness, blueness, and whiteness

**DOI:** 10.1186/s13065-024-01254-8

**Published:** 2024-08-09

**Authors:** Noha S. katamesh, Ahmed Emad F. Abbas, Michael K. Halim, Mohamed A. Abdel-Lateef, Shimaa A. Mahmoud

**Affiliations:** 1 Pharmaceutical Analytical Chemistry Department, Faculty of Pharmacy (Girls), Al-Azhar University, Cairo, 11754 Egypt; 2 Analytical Chemistry Department, Faculty of Pharmacy, October 6 University, 6 October City, Giza 12585 Egypt; 3 Department of Pharmaceutical Analytical Chemistry, Faculty of Pharmacy, Al-Azhar University, Assiut Branch, Assiut, 71524 Egypt

**Keywords:** Montelukast sodium, Fexofenadine hydrochloride, Green micellar UPLC, Eco-friendly spectroscopy, Greenness, blueness and whiteness assessment

## Abstract

The development of sustainable analytical methodologies that minimize hazards, waste generation, and energy consumption has become crucial. This study introduces pioneering green‒blue-white approaches for the simultaneous quantification of montelukast sodium (MLK) and fexofenadine hydrochloride (FEX) in combination formulations. The first approach employs an ultra-performance liquid chromatographic method (UPLC) with a green micellar mobile phase of 0.02 M sodium dodecyl sulfate and 10% 1-pentanol (65:35%). The method demonstrated excellent resolution, peak symmetry, and a short analysis time, with retention times of 3.53 min for MLK and 1.67 min for FEX. The MLK and FEX linearities were 1–260 and 1.2–312 μg/mL, respectively. The second approach involves complementary built-in spectroscopic techniques (second derivative, third derivative, and ratio difference methods) using water as a solvent, providing a green, simple, low-cost alternative in laboratories where expensive chromatographic devices may not be readily available. The MLK and FEX linearities were 3–50 and 3–60 μg/mL, respectively. All methods were comprehensively validated and showed satisfactory results. The proposed methods demonstrated excellent linearity (r^2^ ≥ 0.9990), accuracy (recovery 98.5–101.5%), and precision (RSD ≤ 2%) across wide concentration ranges. A multifaceted evaluation was conducted to assess the environmental sustainability, real-world applicability, and economic viability of the proposed methods in comparison with previously reported techniques. This comprehensive assessment leveraged several state-of-the-art tools, including NEMI, ComplexGAPI, AGREE, ESA, BAGI, and RGB12. The suggested approaches exhibited favorable quadrant profiles in the NEMI and ComplexGAPI assessments, coupled with higher AGREE scores (0.90, 0.86) than reported (0.62, 0.74, 0.75, 0.69, 0.74, 0.74, and 0.75), in addition to higher ESA score (88, 92) than reported (75, 84, 85, 79, 82, 82, and 83), collectively affirming their environmentally friendly credentials. Moreover, we embraced the innovative notions of 'blueness' and 'whiteness' assessment by harnessing the recently formulated BAGI and RGB12 algorithms. The higher BAGI score (90, 82.5) than reported (72.5, 70, 70, 67.5, 67.5, 67.5, and 72.5), confirmed the excellent real-world applicability of the proposed methods, while the notable RGB12 indices (89.8, 88.1) than reported (67.8, 72.8, 71.5, 67.1, 73.7, 70.3, and 73.2), validated their cost-effectiveness and overall sustainability, contributing to an eco-friendly future for quality control processes.

**Supplementary Information:**

The online version contains supplementary material available at 10.1186/s13065-024-01254-8.

## Introduction

Green analytical chemistry (GAC) and white analytical chemistry (WAC) represent transformative shifts in analytical methodologies, advocating for practices that minimize environmental impacts and enhance efficiency throughout the analytical lifecycle [1–5]. GAC prioritizes reducing sample preparation, decreasing energy usage, and eliminating toxic reagents to prevent pollution, enabling waste recycling, and promoting safer chemical production. Similarly, WAC focuses on efficiency, cost-effectiveness, and practicality, guiding the maximization of productivity with minimal resource consumption.

Pharmaceutical quantification, as a cornerstone of analytical endeavors, demands meticulous adherence to these combined green-and-white analytical principles [6–8]. While traditional performance criteria such as accuracy and sensitivity remain crucial, environmental responsibility and judicious resource usage are equally paramount [9]. Solvents such as acetonitrile and methanol, common in conventional methods, conflict with GAC standards due to their appreciable toxicity. Additionally, instruments with prohibitive costs may undermine the practical objectives of WAC [10].

**Fig. 1:**
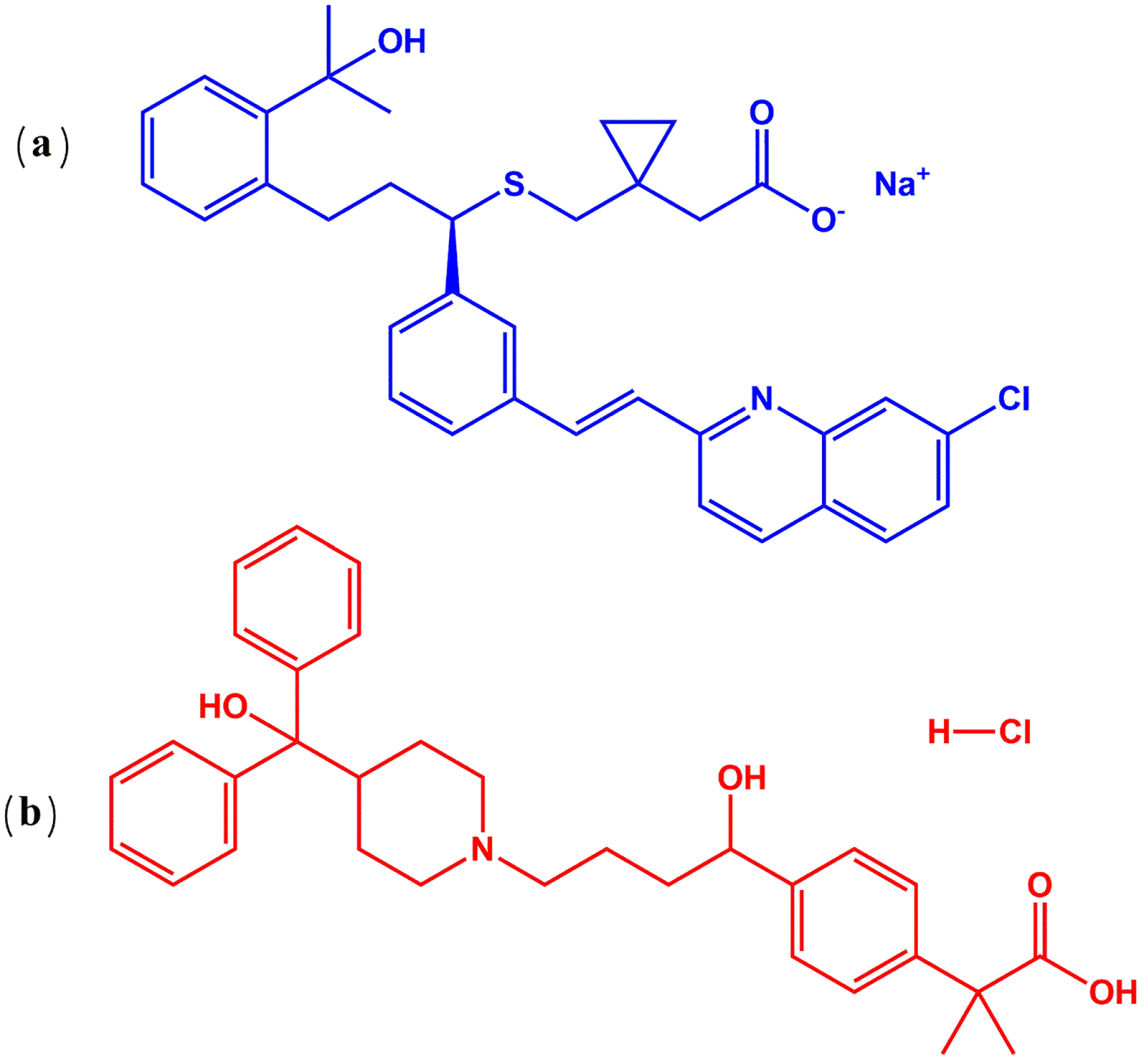
Chemical structure of **a** montelukast sodium, **b** fexofenadine hydrochloride

The significance of developing greener methods is exemplified in the case of montelukast sodium (MLK) and fexofenadine hydrochloride (FEX). Although the combined use of MLK and FEX is widespread and important, existing analytical approaches for this drug combination have significant environmental drawbacks and limitations. Therefore, there is a pressing need to develop newer, greener analytical methods for this combination. MLK, as shown in Fig. 1a, is chemically generated from 2-[1-[[(1*R*)-1-[3-[2-(7-chloroquinolin-2-yl) ethenyl] phenyl]-3- [hydroxypropan-2yl) phenyl] propyl] sulfanylmethyl] cyclopropyl] acetic acid and sodium salt [11]. In 2020, it was ranked as the fourteenth most often prescribed pharmaceutical in the U.S., with a total of more than 31 million prescriptions as a result of its significant role as a potential COVID-19 therapeutic [12]. FEX, as shown in Fig. 1b, is chemically converted to 2-[4[1-Hydroxy-4-[4-(hydroxy-diphenyl methyl)-1-piperidyl] butyl] phenyl]-2-methyl propanoic acid [11]. In 2020, it was ranked as the 255th most commonly prescribed pharmaceutical in the U.S., with a total of more than 1 million prescriptions as a result of the role of FEX as a preventive drug for the treatment of severe acute respiratory syndrome (SARS) because it acts as a potent inhibitor of the main protease of COVID-19 [13]. The concurrent administration of MLK and FEX in combination therapy regimens results in synergistic and complementary effects [14].

Various quantification techniques, including UV‒Vis spectroscopy [15–20], HPLC [21–24], UPLC [25], HPTLC [26], and LC–MS [27], have been established for this drug combination. However, these methods are costly and complex, use nongreen chemicals, and are unsuitable for routine quality control analysis. UV-based methods struggle with specificity issues, particularly low sensitivity and the use of a harmful solvent, methanol, which is not compatible with green chemistry principles due to its potential toxicity and environmental impact. HPLC techniques require run times exceeding 15 min per sample along with substantial volumes of toxic organic solvents such as methanol and acetonitrile. The existing HPTLC protocol, though faster, still relies on unsustainable chlorinated mobile phases. The LC–MS method faces barriers to widespread adoption due to high instrumentation costs and complexity. Compared with other methods, only one published UPLC method offered some improvement in environmental sustainability; however, it still relies on chemicals that are not environmentally friendly, such as acetonitrile. This warrants the development of alternate techniques that align more closely with GAC and WAC principles to balance efficacy and eco-friendliness.

In this study, we aimed to achieve four core objectives. First, we developed a green UPLC method for the quantification of MLK and FEX using a green micellar mobile phase that offers short analysis times, high selectivity, excellent resolution, peak symmetry, and optimal linearity and recovery. Second, develop an eco-friendly complementary spectroscopic approach using water as the solvent for the quantification of MLK and FEX that provides a green, simple, low-cost alternative in laboratories where expensive chromatographic devices may not be readily available. Third, we comprehensively evaluated the greenness of the proposed and reported methods using established tools like the analytical eco-Scale (ESA) tool, the analytical greenness metric (AGREE), the complex complementary green analytical procedure index (ComplexGAPI), and the national environmental method index (NEMI). These ensure environmental benefits compared to reported methods. Fourth, we employed the recently proposed "blueness" and "whiteness" assessment tools, which have been gaining traction in evaluating the practical applicability and overall sustainability of analytical methods, respectively, for all proposed and reported methods, confirming their outstanding performance, dependability, and economic viability. The results for the UPLC method and the three spectrophotometric methods (second derivative, third derivative, and ratio difference) are presented separately. For green assessment tools (AGREE, ESA, NEMI, and ComplexGAPI) and the BAGI tool, the UPLC method is reported, while for spectrophotometric methods, we report one result as there were no differences due to shared principles and eco-friendly solvents and reagents. However, for the RGB12 'whiteness' assessment, considering additional validation parameters, the individual UPLC method score is reported, while for spectrophotometric methods, the average 'whiteness' score across the three methods is presented to account for differences in limits of detection (LOD). Finally, drawing attention to an exemplary model for environmentally friendly analysis without sacrificing accuracy or practical performance exemplifies the commitment to a sustainable future in routine quality control practice. In summary, we draw attention to an exemplary model for environmentally friendly analysis without sacrificing accuracy or practical performance exemplifies the commitment to a sustainable future in routine quality control practice.

## Experimental

### Reagents and materials

The solvents used were of HPLC grade, and all the compounds were of analytical reagent grade. Sodium dodecyl sulfate (SDS, 98.5%), methanol, sodium dihydrogen phosphate, and 1-pentanol were acquired from Sigma‒Aldrich (Steinheim, Germany). Ultrapure water was obtained from a Milli-Q water purification system (Millipore, USA) and used throughout the analysis.

### Samples

#### Reference standards

- The reference standard of MLK (lot no. MK-0180513) with a purity of 99.30% was kindly gifted by EGY Pharm Pharmaceutical Company, Cairo, Egypt.
- The reference standard for FEX (lot no. F007040216, 99.89% purity) was generously provided by Memphis Pharmaceutical Company, Cairo, Egypt.

#### Pharmaceutical sample

The pharmaceutical formulation Montair-FX® tablets (Cipla Ltd., Mumbai, India) was purchased from a local pharmacy. Per the label claim, each tablet was formulated to contain 10 mg of MLK and 120 mg of FEX.

### Instrumentation

- The micellar UPLC system consisted of an Agilent Infinity 1290 model (Agilent Technologies, USA) equipped with a UV detector, a quaternary pump, and a degasser unit DGU-20 (A5). Chromatographic separation was performed on a Kinetex 1.7 μm HILIC 100A column (100 × 2.1 mm, 1.7 μm particle size) (Phenomenex, USA) maintained at 40 °C in a column oven.
- Absorbance measurements were made on a Shimadzu UV-1800 spectrophotometer (Shimadzu Corp., Japan) with 1 cm path length quartz cuvettes. The spectrophotometer was operated using in-built UVProbe software version 2.21 with a scanning speed of 2800 nm/min and bandwidth of 2 nm.

### Standard solutions

Primary stock solutions of MLK and FEX (1 mg/mL for UPLC, 100 μg/mL for spectrophotometry) were prepared by dissolving reference standards in ultrapure water. Working standard solutions were freshly prepared by appropriate dilutions of stock solutions with ultrapure water to yield the concentrations required for constructing calibration curves. The prepared solutions were stable when refrigerated at 4 °C for 1 week.

### Procedures

#### Micellar UPLC method

**Fig. 2:**
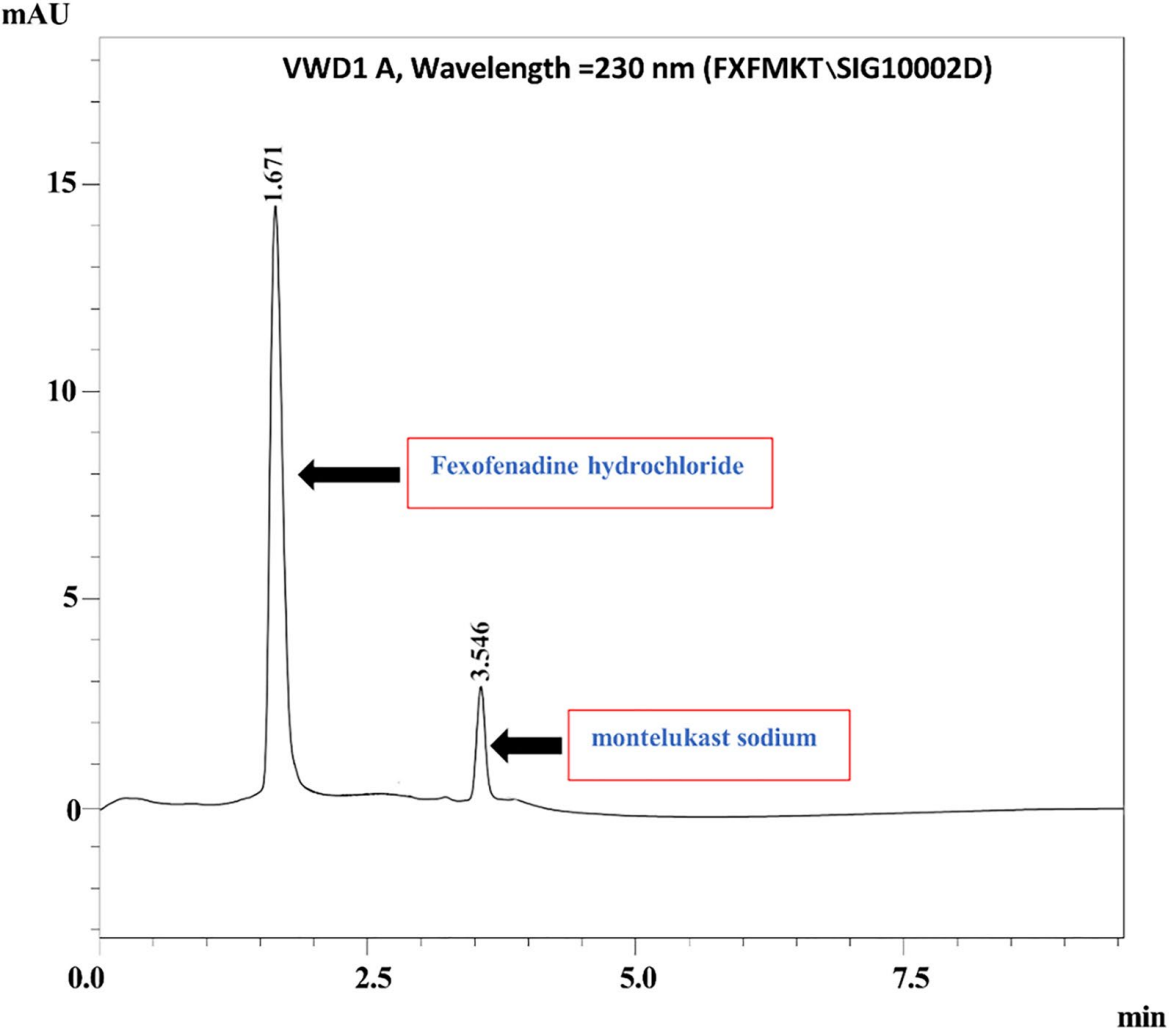
Micellar UPLC chromatogram demonstrating the separation of 4 μg/mL MLK and 48 μg/mL FEX from the laboratory-prepared mixture

The micellar mobile phase consisted of 0.02 M SDS and 10% 1-pentanol (65:35%) buffered at pH 3.5 with 0.05 M sodium dihydrogen phosphate. The mobile phase was filtered through a 0.22 μm membrane filter and degassed ultrasonically prior to use. The flow rate, column temperature, and detection wavelength were maintained at 1 mL/min, 40 °C, and 230 nm, respectively. Under optimized conditions, the MLK and FEX peaks appeared at retention times of 1.67 and 3.54 min, respectively, as shown in Fig. 2.

#### Spectroscopic methods

**Fig. 3:**
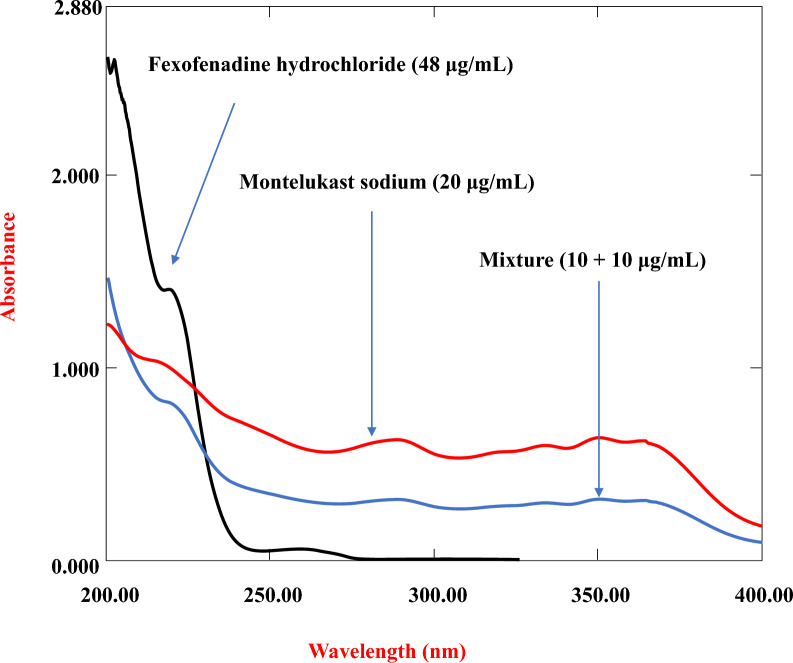
Zero-order spectra of MLK and FEX and a mixture of MLK and FEX in distilled water

UV‒Vis spectra of standard MLK and FEX solutions were acquired from 200–400 nm using water as a blank, and the overlaid zero-order absorption spectra are presented in Fig. 3.

### Construction of calibration curves

#### UPLC method

Appropriate volumes of standard drug solutions were diluted with the mobile phase to yield concentrations in the range of 1–260 μg/mL for MLK and 1.2–312 μg/mL for FEX. The solutions were subsequently examined by injection into the chromatographic system. The calibration plots were generated by plotting the peak area versus the corresponding concentration in μg/mL, after which the relevant regression equation was calculated.

#### Spectroscopic methods

A series of 10 mL volumetric flasks were prepared with aliquots of standard MLK and FEX solutions ranging from 3–50 μg/mL for MLK and 3–60 μg/mL for MLK and FEX, respectively, and then filled to the required volume with water. The absorption spectra of the prepared drug solutions were measured at 200–400 nm using water as a blank.

##### Second derivative method (^2^D)

**Fig. 4:**
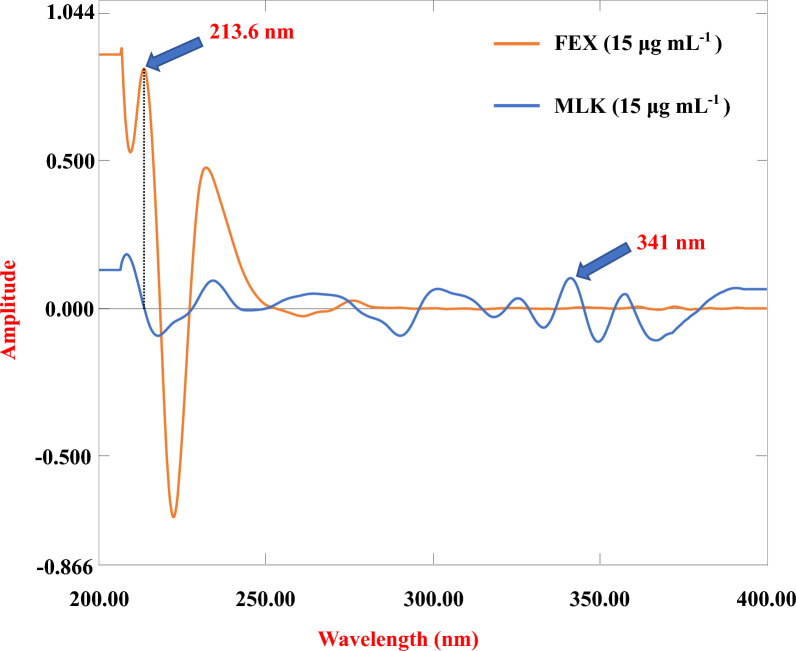
Second-order spectra of MLK and FEX

The stored spectral data were processed to obtain ^2^D spectra using built-in UV-Probe software with a wavelength difference (Δλ) = 8 nm and a scaling factor (SF) = 150. The peak amplitudes at 341 nm for MLK and 213.6 nm for FEX were measured. Calibration plots were generated by graphically representing the peak amplitudes against their respective concentrations expressed in μg/mL, as illustrated in Fig. 4, and the pertinent regression equation was computed.

##### Third derivative method (^3^D)

**Fig. 5:**
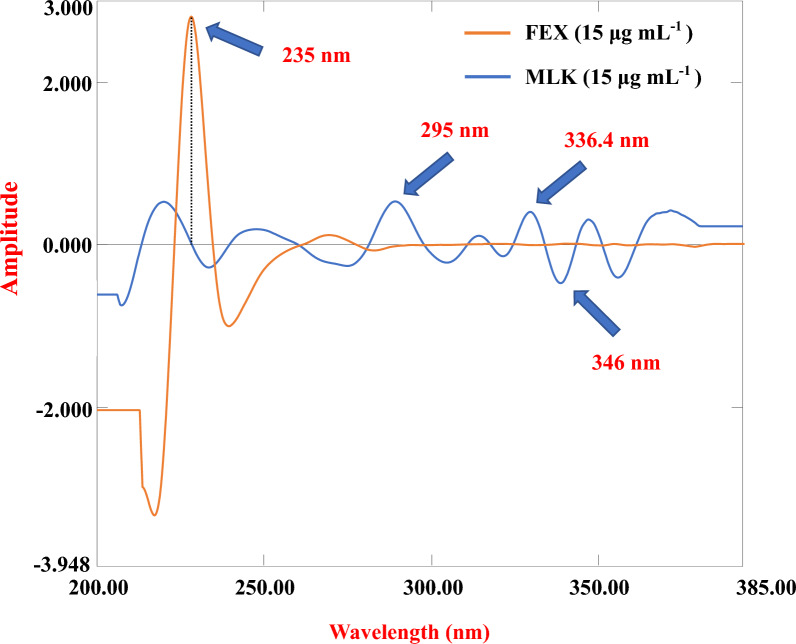
Third-order spectra of MLK and FEX

The ^3^D spectra were obtained from the stored data using built-in UV-Probe software with Δλ = 16 nm and SF = 6000. The peak amplitudes at 295, 336.4, and 346 nm for MLK and at 235 nm for FEX were measured. Calibration plots were generated by graphically representing the peak amplitudes against their respective concentrations expressed in μg/mL, as illustrated in Fig. 5, and the pertinent regression equation was computed.

##### Ratio difference (RD) method

The ratio spectra of MLK were acquired by dividing the recorded MLK's zero-order absorption spectra by the FEX divisor spectrum (15 μg/mL). Similarly, the FEX ratio spectra were acquired by dividing the recorded FEX zero-order absorption spectra by the MLK divisor spectrum (5 μg/mL). Calibration curves were constructed by plotting the difference in amplitudes at 250 and 245 nm for MLK or 227 and 217 nm for FEX versus the corresponding concentrations, as shown in Fig. S1, and the relevant regression equation was calculated.

#### Analysis of laboratory-prepared mixtures

- UPLC method
  Aliquots of MLK and FEX were added to 10 mL volumetric flasks, which were then filled with mobile phase to yield different concentration ratios of the two drugs covering the linear range. The solutions were mixed well and analyzed under optimized UPLC conditions. The concentrations of MLK and FEX were determined from the respective regression equations.
- Spectroscopic method
  Aliquots of MLK and FEX were added to 10 mL volumetric flasks, which were then filled with water to yield different concentration ratios of the two drugs. The absorption spectra of these laboratory mixtures were measured and recorded as described earlier under linear conditions. The concentrations of MLK and FEX were determined using the respective spectroscopic methods.

#### Pharmaceutical dosage form analysis

The proposed methods were applied to the quantitative determination of MLK and FEX in their combined tablet formulation. Five Montair FX® tablets, each labeled with 10 mg of MLK and 120 mg of FEX, were collectively pulverized into a fine powder. An accurately weighed amount of the powdered tablets, equivalent to 10 mg of MIK and 120 mg of FEX, was transferred into a 100 mL volumetric flask. Approximately 50 mL of water was introduced into the flask, and the mixture was subjected to sonication for 15 min to facilitate the extraction of the active pharmaceutical ingredients into the aqueous medium. The solution was then diluted to the final volume with water, thoroughly mixed, and filtered through a 0.45 μm membrane filter. Subsequent dilutions with water yielded final concentrations of 10 μg/mL for MLK and 120 μg/mL for FEX. Aliquots from the clear filtrate were placed into 10 mL volumetric flasks and further diluted with water. The established procedures, as detailed previously, were meticulously followed, and regression equations were employed to determine the concentrations of MLK and FEX in the tablet samples.

## Results and discussion

The pursuit of analytical methodologies that harmonize sustainability principles with high performance remains a pressing challenge [8]. Consequently, the overarching objective of this study was to devise an innovative and environmentally conscious approach for the simultaneous quantification of the anti-COVID pharmaceuticals MLK and FEX. This was achieved through the strategic integration of selective micellar UPLC and complementary spectroscopic techniques that leveraged highly sensitive detection and eco-friendly solvents. The strategic integration of UPLC technology and eco-friendly micellar liquid chromatography provides an optimal model for balancing separation efficiency with green chemistry aspirations [28]. Specifically, the UPLC column provides exceptional peak resolution, sensitivity, and symmetry within a drastically shortened run time of only 3.5 min, enhancing sample throughput. However, using that technique along with commonly used but environmentally harmful liquid solvents like acetonitrile or methanol can threaten sustainability efforts. Therefore, the introduction of a biocompatible micellar mobile phase containing the surfactant SDS and pentanol modifier mitigates this limitation, avoiding nearly all toxic solvent use. In liquid chromatography (LC), the mobile phase typically consists of organic solvents, such as acetonitrile or methanol, mixed with water. These organic solvents are often toxic and environmentally harmful. Micellar liquid chromatography [29], on the other hand, uses an aqueous solution of a surfactant, such as SDS, as the mobile phase. In aqueous solutions above a certain concentration (known as the critical micelle concentration, CMC), SDS molecules self-assemble into colloidal aggregates called micelles. These micelles have a hydrophobic core and a hydrophilic outer shell, which can mimic the behavior of organic solvents in LC. The hydrophobic cores of the micelles can accommodate non-polar analytes, while the polar outer shells can interact with polar analytes, facilitating their partitioning and subsequent separation. When an analyte is introduced into the micellar mobile phase, it can partition between the aqueous phase and the hydrophobic core of the micelles, similar to how it would partition between the aqueous and organic phases in RP-HPLC. This partitioning behavior enables the separation of analytes based on their hydrophobicity, just like in LC. This micellar UPLC approach thereby maximizes chromatographic performance while manifesting the green analytical principles of benign-by-design chemicals and waste minimization.

Likewise, transitioning to complementary spectroscopic techniques introduces a practical, low-cost analytical alternative that further reduces solvent consumption by employing only water [30]. With accurate, precise measurements rivaling complex assays, these sustainable spectroscopic methods exemplify another green chemistry model reconciling efficacy and environmentally responsible practices, which is particularly valuable for modest laboratories lacking expensive instrumentation. Moreover, the methods presented in this study involved a multifaceted evaluation process employing an array of complementary sustainability assessment techniques. These comprehensive assessments aimed to scrutinize the green and white credentials of the proposed approaches. The results emerging from these evaluations provided compelling evidence of the remarkable alignment of the recommended strategies with the established parameters that define sustainable analytical chemistry practices.

### UPLC method development and optimization

The most critical chromatographic parameters affecting MLK and FEX separation were investigated and analyzed carefully to achieve satisfactory separation of the two drugs with symmetrical, sharp peak shapes within a minimal duration. The ICH guidelines [31] describe the calculation of chromatographic performance in terms of the number of theoretical plates, resolution, and tailing factor, and the results are presented in Table S1.

#### Selection of columns

To reduce solvent consumption and develop a greener method, we evaluated columns with shorter lengths, smaller internal diameters, and smaller particle sizes. Several reversed-phase columns, including Kinetex® HILIC (100 × 2.1 mm, 1.7 μm), Kinetex C18 (100 × 4.6 mm, 2.6 μm), Zorbax Eclipse XDB-C18 (150 × 4.6 mm, 5 μm), and Monolithic RP-C18 (100 × 4.6 mm), were tested for the optimal separation of MLK and FEX.

Through these trials, the Kinetex HILIC column emerged as the most suitable choice, providing symmetrical peaks and the shortest analysis time of 3.5 min. The superior performance of this column can be attributed to its favorable interactions with the chemical and physical properties of MLK and FEX [32].

Both MLK and FEX exhibit moderate polarity, with topological polar surface areas (TPSA) of 73.25 Å^2^ and 81 Å^2^, respectively, arising from the presence of hydroxyl groups, a piperidine ring (in FEX), and other polar functional groups. The HILIC (Hydrophilic Interaction Liquid Chromatography) stationary phase is specifically designed to separate polar and ionizable compounds through favorable interactions with these polar groups, contributing to the efficient separation observed for MLK and FEX.

Additionally, the pKa values of MLK (strongest basic pKa of 3.49) and FEX (strongest basic pKa of 9.21) indicate their potential to exist as cations under certain pH conditions. The HILIC mechanism involves electrostatic interactions between the analytes and the stationary phase, which can contribute to the separation based on the ionization behavior of these compounds.

Furthermore, the relatively large molecular sizes of MLK (608.17 g/mol) and FEX (501.67 g/mol), along with their complex structures, are accommodated by the 100 Å pore size of the Kinetex HILIC column, facilitating their retention and separation. Although the HILIC mechanism primarily relies on polar interactions, the presence of hydrophobic regions in MLK (logP 8.421) and FEX (logP 2.939) can contribute to additional interactions with the stationary phase, potentially enhancing their retention and selectivity.

In contrast, the other evaluated columns, such as Kinetex C18, Zorbax Eclipse XDB-C18, and Monolithic RP-C18, exhibited broader peaks, longer retention times, and insufficient resolution, as shown in Fig S2. These columns may not have provided optimal separation conditions for MLK and FEX based on their chemical and physical properties.

#### Selection of detection wavelength

Various wavelengths, including 200, 210, 220, 230, 240, 250, and 260 nm, were tested. Based on spectral evaluation and the observation of a better baseline, 230 nm was selected as the optimal detection wavelength, as shown in Fig S3. The choice of 230 nm is scientifically justified by the fact that both MLK and FEX exhibit their maximum UV absorption around this wavelength due to the presence of chromophoric groups in their structures. This maximum absorption ensures the highest sensitivity during quantification, as it aligns with the optimal energy level for the molecular interaction forces of these compounds.

#### Optimization of the mobile phase

Including improved safety, cost-effectiveness, and eco-friendliness. Micelles, colloidal aggregates of surfactants, demonstrate excellent chromatographic efficiency. Additionally, micellar mobile phases enable greener analysis by minimizing the use of toxic organic solvents.

To achieve optimal separation of MLK and FEX within a reasonable time frame, various mobile phase compositions were evaluated through trials. The impact of varying the concentration of SDSs over the range of 0.01–0.04 M on the retention times and separation of the drugs was investigated (Table S1). The most effective resolution was obtained with 0.02 M SDS.

Furthermore, different organic modifiers, including methanol, acetonitrile, ethanol, n-propanol, n-butanol, and n-pentanol, were tested to enhance the separation (Fig S4). When ethanol and methanol were used, no separation occurred for MLK and FEX, indicating their incompatibility with the chemical properties of these drugs. The use of *n*-butanol, acetonitrile, or n-propanol resulted in broad peaks with long retention times, suggesting suboptimal interactions with the analytes.

Additionally, the effect of varying the percentage of 1-pentanol from 5 to 20% was studied. It was found that incorporating 15% 1-pentanol in the mobile phase yielded satisfactory resolution, symmetrical peaks, and good peak clarity within a reasonable analysis time (Table S1).

The selection of 0.02 M SDS and 15% 1-pentanol as the optimal mobile phase composition is scientifically justified by the chemical and physical properties of MLK and FEX. Both drugs possess moderate polarity, as indicated by their topological polar surface areas (TPSA) of 73.25 Å^2^ for MLK and 81 Å^2^ for FEX, arising from the presence of hydroxyl groups, a piperidine ring (in FEX), and other polar functional groups. The micellar mobile phase, comprising SDS and the pentanol modifier, can effectively interact with these polar moieties, facilitating efficient separation.

Moreover, the pKa values of MLK (strongest basic pKa of 3.49) and FEX (strongest basic pKa of 9.21) indicate their potential to exist as cations under certain pH conditions. The micellar mobile phase can engage in electrostatic interactions with the ionized forms of these compounds, further contributing to their separation based on their ionization behavior.

Furthermore, the relatively large molecular sizes of MLK (608.17 g/mol) and FEX (501.67 g/mol), along with their complex structures, are accommodated by the micellar mobile phase, enabling their retention and separation. Although the micellar mechanism primarily relies on polar interactions, the presence of hydrophobic regions in MLK (logP 8.421) and FEX (logP 2.939) can contribute to additional interactions with the micellar system, potentially enhancing their retention and selectivity.

#### Effects of pH and buffer concentration

The effect of pH on the chromatographic behavior of MLK and FEX was investigated over the range of pH 3.0 to 4.5 using 0.05 M sodium dihydrogen phosphate buffer in the mobile phase. A pH of 3.5 was found to provide maximum theoretical plates and optimal peak symmetry for both analytes.

Additionally, the impact of varying buffer concentrations was evaluated. Among the tested concentrations, 0.05 M buffer led to well-resolved and sharp peak shapes, as evident from the sample chromatogram (Table S1). Consequently, a 0.05 M buffer at pH 3.5 was selected as the final mobile phase condition.

The selection of pH 3.5 is scientifically justified by the ionization behavior of MLK and FEX, as dictated by their pKa values. At this pH, MLK (strongest basic pKa of 3.49) is likely to exist in a partially ionized form, while FEX (strongest basic pKa of 9.21) remains predominantly neutral. The optimal peak shapes and resolution achieved at pH 3.5 suggest that this condition facilitates favorable interactions between the analytes and the micellar mobile phase, contributing to their efficient separation.

#### Effects of flow rate and column temperature

The flow rate was varied from 0.6 to 1.2 mL/min during the optimization process. A flow rate of 1 mL/min was found to be optimal, as it provided better peak shapes, shorter retention times for both analytes and satisfactory peak resolution.

Similarly, the column oven temperature was varied from 30 °C to 50 °C. While higher temperatures increased the theoretical plates, they also proportionately increased peak tailing. To achieve the best peak shapes and resolution, a column temperature of 40 °C was maintained (Table S1).

The selection of a 1 mL/min flow rate and a column temperature of 40 °C is scientifically justified based on the principles of chromatographic separation and the physical properties of MLK and FEX.

The flow rate directly influences the linear velocity of the mobile phase through the column, affecting the mass transfer kinetics and the equilibration time between the stationary and mobile phases. A flow rate of 1 mL/min likely provides an optimal balance between efficient mass transfer and reasonable analysis time, contributing to the observed peak shapes and resolution.

The column temperature, on the other hand, affects the viscosity of the mobile phase, the diffusion coefficients of the analytes, and the interactions between the analytes and the stationary phase. At 40 °C, the mobile phase viscosity and analyte diffusion rates are likely optimized, facilitating efficient mass transfer and minimizing peak tailing. Additionally, the interactions between MLK, FEX, and the micellar mobile phase may be optimal at this temperature, contributing to the observed peak shapes and resolution.

### Spectrophotometric methods

The overlaid UV absorption spectra of MLK and FEX (Fig. 3) showed a severe overlap, making direct simultaneous estimation difficult. Hence, advanced spectroscopic techniques were developed for their quantification. Three simple, economical, and accurate derivative (^2^D, and ^3^D) and ratio difference (RD) spectrophotometric methods were developed.

#### Development and optimization

##### Selection of solvent

The absorption intensity and solubility of the MLK and FEX standards were studied in distilled water, ethanol, acetonitrile, methanol, and aqueous buffers of varying pH, such as 0.1 M HCl, pH 8 borate buffer, 0.1 M NaOH, and pH 4 acetate buffer. Distilled water gave the maximum absorption intensity and solubility for both drugs and was selected as a blank/solvent for eco-friendly analysis.

##### Delta lambda and scaling factor for the methods

For derivative spectrophotometry, the derivative order, Δλ, and SF concentration were optimized to achieve sufficient sensitivity and selectivity at working concentrations. The initial assessment involved calculating the first derivative of the ratio spectra, but no clear peaks were identified. Consequently, the second and third derivatives of the ratio spectra were employed. For the ^2^D method, parameters of Δλ = 8 nm and SF = 150 gave well-defined peaks with the best signal-to-noise ratio. Similarly, for ^3^D, Δλ = 16 nm and SF = 6000 were optimal.

##### Selection of divisors

Different concentrations of FEX and MLK standards were evaluated as divisors to determine the optimal RD method. FEX at concentrations of 15 μg/mL and MLK at 5 μg/mL gave the best recovery with the highest sensitivity and reproducibility.

#### Method characteristics

##### Second derivative method

This technique involves the conversion of a normal zero-order absorption spectrum to a ^2^D spectrum [30]. This approach helps eliminate spectral interference by abolishing background effects and improving detection selectivity. In this method, the zero-order absorption spectra of FEX and MLK were transformed into ^2^D signals. Then, each was determined at a zero-crossing point where the other had no absorbance at this point. In the present work, ^2^D spectra of MLK and FEX were obtained by UV-Probe software using Δλ = 8 nm and SF = 150. The amplitudes of MLK and FEX at their zero crossing points of 341 nm and 213.6 nm, respectively, were used for quantitation, as shown in Fig. 4.

##### Third derivative method

This technique involves the conversion of a normal zero-order absorption spectrum to a ^3^D spectrum [30]. The zero-crossing point in the derivative spectrum corresponds to the λ_max_ of a compound, permitting direct estimation without interference. Here, ^3^D spectra were obtained using Δλ = 16 nm and SF = 6000. The peak amplitudes at 295, 336.4, and 346 nm for MLK and 235 nm for FEX showed good linearity and were used for the assay, as shown in Fig. 5.

##### Ratio difference method

In this method, the ratio spectra were obtained by dividing the standard spectra of one analyte by a fixed concentration of the other analyte as a divisor [30]. The difference in amplitudes at two wavelengths was proportional to the analyte concentration but was zero for the divisor. For MLK, the difference between 250 and 245 nm, and for FEX, the difference between 227 and 217 nm showed good linearity and sensitivity and were used for the assay, as shown in Fig. S1.

### Method validation

The proposed micellar UPLC and spectroscopic methods were validated as per the ICH recommendations [31].

#### System suitability testing

To evaluate system performance, system suitability parameters such as retention period (t_R_), number of theoretical plates (N), and resolution (R_s_) were investigated as system appropriateness criteria. The optimized chromatographic conditions yielded an exceptional baseline resolution of the two analytes with negligible peak tailing. The chromatographic peaks exhibited high symmetry, ensuring accurate and precise quantification, as indicated in Table S2.

#### Linearity range

UPLC calibration plots were meticulously established for MLK concentrations ranging from 1–260 µg/mL and FEX concentrations from 1.2–312 µg/mL. For spectroscopic methods, MLK had a linearity range of 3–50 µg/mL and FEX had 3–60 µg/mL. Table 1 summarizes calculated regression characteristics. These high correlation values confirm that drug concentrations and analytical responses are linearly related.

**Table 1:** The regression coefficients and validation outcomes about the quantification of MLK and FEX employing the proposed methodological approaches

Parameter	Micellar UPLC method		Spectroscopic methods							
			Second derivative (^2^D)		Third derivative (^3^D)				Ratio Difference (RD)	
	MLK	FEX	MLK	FEX	MLK			FEX	MLK	FEX
Wavelength (nm)	230	230	341	213.6	295	336.4	346	235	ΔP 250–245	ΔP 227–217
Linearity range (μg/mL)	1–260	1.2–312	3–50	3–60	3–50	3–50	3–50	3–60	3–50	3–60
Slope	9.672 ±0.11	0.4582 ± 0.002	0.0109 ± 0.0027	0.0355 ± 0.0002	0.0513 ± 0.0003	0.039 ± 0.0002	0.0482 ± 0.0002	0.0391 ± 0.0003	0.0509 ± 0.0004	0.0346 ± 0.0003
Intercept	3.317 ± 0.028	4.281 ± 0.26	− 0.0041 ± 0.0001	0.1905 ± 0.0072	− 0.0362 ± 0.0091	0.0105 ± 0.0054	0.0698 ± 0.0068	0.0356 ± 0.0092	− 0.0544 ± 0.0125	0.0314 ± 0.0089
Determination coefficient (r^2^)	0.999	0.999	0.9996	0.9998	0.9998	0.9999	0.9999	0.9998	0.9996	0.9997
Accuracy (%R)	99.67	98.94	100.56	99.54	101.28	100.91	100.27	98.62	99.37	101.82
Precision (%RSD):										
Repeatability	1.53	1.43	0.36	0.54	0.62	0.71	0.68	1.06	0.64	0.52
Intermediate precision	0.23	1.16	0.34	1.37	0.85	0.87	0.91	0.54	0.83	1.16
LOD (μg/mL)	0.452	0.079	0.828	0.6721	0.588	0.456	0.467	0.778	0.811	0.853
LOQ (μg/mL)	1.37	0.264	2.508	2.037	1.781	1.382	1.414	2.357	2.458	2.586

#### Sensitivity

Following the guidelines set forth by the ICH [31], the minimum concentrations required for reliable detection (limits of detection, LOD) and quantification (limits of quantification, LOQ) were calculated. The low LOD and LOQ values indicate the adequate sensitivity of the proposed methods for precisely detecting and quantifying MLK and FEX, as shown in Table 1.

#### Accuracy and precision

Nine distinct measurements were taken at three concentrations (5, 10, and 20 μg/mL) for MLK and 60, 120, and 220 μg/mL for FEX via the micellar UPLC method, while spectroscopic methods were used (10, 20, and 50 µg/mL) and 6, 24, and 48 μg/mL) to assess the accuracy of the MLK and FEX methods, respectively, as shown in Table 1. To evaluate the precision of the experiment, its repeatability and intermediate precision were examined, as shown in Table 1.

#### Selectivity

By testing various laboratory-prepared mixtures containing MLK and FEX separately within the linear range, the selectivity of the techniques was determined, and promising findings were achieved. Acceptable results were achieved and can be found in Table S3.

#### Robustness

The method's robustness was assessed by investigating the impact of slight modifications in the mobile phase's pH (± 0.1), flow rate (± 0.1), and concentration (± 0.1%). No discernible impact was found.

#### Application to tablet preparation

Using the provided techniques, the drug content of Montair-FX® tablets was successfully estimated. The results met expectations and showed excellent accuracy with respect to the amount claimed on the label, with no evidence of excipient interference. Additionally, the F test, Student's t-test, and one-way ANOVA were used with a 95% confidence level to compare the results with those from the reported methodology [25] statistically, there were no appreciable differences between the suggested and reported methods, as shown in Tables 2 and 3.

**Table 2:** Comparative statistical evaluation of results obtained by the proposed and the reported methods for the analysis of MLK and FEX

Parameters	Micellar UPLC method		Spectroscopic methods								Reported method [25]	
			Second derivative		Third derivative				Ratio difference			
	MLK	FEX	MLK	FEX	MLK			FEX	MLK	FEX	MLK	FEX
Linearity range (μg/mL)	1–260	1.2–312	3–50	3–60	3–50 (295 nm)	3–50 (336.4 nm)	3–50 (346 nm)	3–60 (235 nm)	3–50	3–60	12.5–37.5	150–450
n	5	5	5	5	5	5	5	5	5	5	5	5
Mean %	99.74	100.04	99.77	100.15	100.59	100.01	100.41	99.31	99.76	100.52	100.18	100.32
SD	1.29	1.36	1.04	1.05	0.79	0.91	1.23	0.60	0.79	0.84	1.21	0.79
variance	1.66	1.76	1.08	1.10	0.62	0.82	1.51	0.36	0.62	0.70	1.47	0.627
Student’s t test (2.306)	0.48	0.41	0.57	0.28	0.64	0.25	0.30	2.27	0.65	0.40	–	–
F value (6.39)	1.73	2.58	1.35	1.76	2.33	1.76	1.03	1.76	2.38	1.11	–	–

**Table 3:** One-way ANOVA for comparative evaluation of proposed and literature-reported methodologies in pharmaceutical formulation analysis with a 95% confidence interval

Component		Sum of squares	df	Mean square	F	P value
MLK	Between Groups	5.275	4.000	1.319	0.838 (2.866)^a^	0.517
	Within Groups	31.466	20.000	1.573	–	–
	Total	36.741	24.000	–	–	–
FEX	Between Groups	9.341	4.000	2.335	2.100 (2.866)^a^	0.119
	Within Groups	22.240	20.000	1.112	–	–
	Total	31.580	24.000	–	–	–

^a^Figures in parentheses represent the corresponding critical value of F at P < 0.05

### Comparison of calibration curve construction methods

UPLC and three spectroscopic methods (2D, 3D, and RD) were used. Direct side-by-side comparisons of key validation parameters shed light on relative performance trade-offs to help choose methods.

Spectral interferences are eliminated by selective detection at specific retention times with UPLC chromatographic separation. Thus, 230 nm was chosen to maximize MLK and FEX absorbance and sensitivity without matrix interference. However, spectral overlap hinders direct UV estimation. By computationally removing background signals, derivative methods solve this problem. Each derivative order has mathematical properties specific to analytes and matrices. In 2D, MLK and FEX peak amplitudes at 341 and 213.6 nm were calibrated. In 3D, MLK peak amplitudes at 295, 336.4, and 346 nm were proportional to concentration. For estimation, the 235 nm FEX peak was used. Finally, the RD is divided by the standard analyte spectrum to reduce interference. The defined absorbance ratios varied with concentration. MLK used 250 nm to 245 nm amplitude difference, and FEX used 227 nm to 217 nm.

UPLC had the widest linear concentration range, allowing for maximum calibration flexibility. Due to detector response constraints at higher concentrations, all spectrophotometric methods had narrower ranges. UPLC showed that FEX (1**.**2–312 μg/mL) had a wider linear range than MLK (1–260 μg/mL), suggesting stronger hydrophilic and H-bonding interactions with the stationary phase. Due to its stronger retention, FEX elutes later than MLK under chromatographic conditions. FEX can be quantified over a wider linear range before peak broadening or distortion due to its higher retention and column capacity factor. In contrast, MLK has a lower column capacity and weaker retention. To prevent peak broadening, which could compromise quantitation accuracy, MLK's linear range was narrowed to lower concentrations. Spectroscopic techniques yielded narrower linearity ranges of 3–50 µg/mL for MLK and 3–60 µg/mL for FEX. As simpler instrumentation has slightly higher detection limits, the linear ranges reach higher lower limits. The upper ends stay within Beer's law for the cuvette path length. The derivatization and ratio difference processes in the spectroscopic methods reduce background noise and improve analytical sensitivity, bringing MLK's linear range closer to FEX. In contrast, UPLC uses only intact drug molecules' native UV absorbance. Thus, the inherent sensitivity difference remains. This method boosts MLK sensitivity.

In terms of sensitivity, UPLC found 3–fourfold lower LODs for FEX, reaching 0.0079 µg/mL, almost 10 times the initial targeted concentration. Optimized chromatographic separation methods can quantify ultra-trace amounts. Moreover, spectrophotometric methods produced comparable LODs (0.5–0.8 μg/mL) suitable for pharmaceutical analysis. UPLC's baseline resolution, peak symmetry, and efficient separation in a packed column with small particles increase detection sensitivity. This quantifies lower drug concentrations than cuvette spectroscopy. The UV extinction coefficients of MLK and FEX molecular structures affect UPLC detector response factors. FEX outperforms MLK at 230 nm in UV absorption and detector signals at equal concentrations. This explains why FEX had a fivefold lower LOD (0.0079 μg/mL) than MLK (0.452 μg/mL) using the same UPLC instrumentation.

All methods had consistent accuracies and precisions between 98 and 102% and RSD values within 1.53%. This proves the proposed method's quantitative performance meets the acceptance criteria. These methods validate core data but have strengths and weaknesses. UPLC separation resolves structurally similar components with unmatched selectivity. It also detects individual analytes in complex matrices using multi-wavelength analyses. UPLC requires specialized infrastructure and vast method development expertise. Compared to other methods, spectrophotometry is simple and cost-effective, utilizing UV–visible instruments commonly found in labs. However, spectral signal decomposition requires complex mathematical calculations due to separation capacity limitations.

Overall, while all the presented calibration methods show fit-for-purpose validation characteristics, researchers must consider infrastructure constraints and specific quantification needs when selecting optimal techniques. UPLC offers superior flexibility and sensitivity but requires liquid chromatographic systems. Moreover, derivative and ratio spectrophotometry provides affordable, accessible alternatives, albeit with narrower ranges. Thus, balancing key trade-offs can guide sustainable analytical choices.

### Comprehensive greenness, blueness, and whiteness evaluation

Analytical methods' sustainability credentials must be evaluated since they offer priceless information about their possible environmental and economic effects. But the idea of sustainability is complex and includes many different aspects, such as cost-effectiveness, safety concerns, waste reduction techniques, and environmental responsibility (greenness) [8]. No single tool or assessment method can evaluate sustainability across all relevant parameters [30]. This study uses a synergistic multi-tool approach to address this challenge by integrating complementary assessment strategies from different perspectives. This strategic combination of evaluation methods allows a more holistic and robust assessment of the analytical techniques' sustainability.

#### Evaluation of the method's greenness profile

##### NEMI criteria

The NEMI analysis offered an initial screening of greenness deficiencies through a visual format separated into four quadrants [33]. One PBT (persistent, bioaccumulative, and toxic), two hazardous, three corrosive, and four waste products are the contents of these four quadrants. The green quadrant signifies the following: (1) the reagents used are not PBT-classified according to the Toxic Release Inventory (EPA-TRI) of the Environment Protection Agency; (2) the employed substances are deemed nonhazardous and are thus not included in the TRI list; and (3) the pH of the medium is between 2 and 12. Additionally, waste generation is less than 50 g (Fig. S5). For the suggested methodology, NEMI pictograms were generated **(**Tables 4 and 5**)**. A preliminary examination of the pictograms revealed that the recommended method satisfied all the NEMI criteria, as evidenced by the four green quadrants.

**Table 4:** Comparative study of the proposed and reported UPLC methods

Parameter	Proposed method	Reported method [25]
NEMI tool		
ESA tool	88	75
Complex GAPI tool		
AGREE tool		
BAGI tool		`
RGB 12 algorithm

**Table 5:** Comparative study of the proposed and reported spectrophotometric methods

Parameter	NEMI tool	ESA tool	ComplexGAPI tool	AGREE tool	BAGI tool	RGB 12 algorithm
Proposed method		92				
Reported method [15]		84				
Reported method [16]		85				
Reported method [17]		79				
Reported method [18]		82				
Reported method [19]		82				
Reported method [20]		83				

##### ComplexGAPI criteria

NEMI allows introductory greenness examination, but Complex GAPI allows a more comprehensive semi-quantitative evaluation [34]. It is better than the original GAPI measure because it includes a hexagon-shaped area showing pre-analysis phases and stages, as illustrated in Fig. S6. This advanced tool handles all steps of the analytical process, from collecting and transporting samples to securing, storing, preparing, and analyzing them [34]. Complex GAPI offers easy-to-use pictogram software. Green icons and a low E-factor value show that this study's methodologies are very green. With an impressive E factor of one, these methods generate minimal waste, improving sustainability and the environment. This remarkable accomplishment underscores the superiority of the developed approaches over the reported techniques in terms of greenness, as summarized in Tables 4 and 5. Despite its benefits, ComplexGAPI's scope is limited to environmental criteria, leaving waste prevention, energy efficiency, and renewable resource incorporation unaddressed. Overreliance on ComplexGAPI alone may result in an incomplete and narrow assessment. Complementing ComplexGAPI analysis with quantitative assessment methods can overcome this limitation and provide a more complete evaluation. ComplexGAPI and other complementary tools will bridge research gaps and address the constraints of using a single assessment technique in this integrated approach.

##### AGREE criteria

The AGREE metric, which takes into account all 12 GAC philosophies, is a beneficial quantitative method for measuring greenness [35]. This enables thorough assessment grounded in widely accepted GAC criteria. A key advantage of AGREE is its flexibility through customizable weighting of these diverse parameters. The user-friendly software converts the 12 inputs into a single score from 0 to 1, visualized on a colored pictogram for rapid interpretation. Dark green indicates excellent greenness, while dark red signifies major deficiencies. For this study, the proposed methodologies exhibited exemplary greenness, attaining impressive scores of 0.86 and 0.9 for the UPLC and spectrophotometric techniques, respectively underscores the superiority of the developed approaches over the reported techniques, as shown in Figs. S7–S15. These scores signify their efficient alignment with green principles. Visualizations in Tables 4 and 5 show how environmentally friendly the proposed methods are, proving that they follow sustainable analytical practices. Since the AGREE tool only evaluates environmental criteria related to green chemistry principles, it ignores safety, analytical performance, and cost-effectiveness assessments. Thus, AGREE alone may provide an incomplete and narrow evaluation. AGREE should be used with complementary tools that evaluate these additional sustainability aspects to overcome these limitations and ensure a complete assessment.

##### ESA criteria

The ESA is a widely employed green metric for assessing the environmental impact of analytical procedures. It offers several advantages over other assessment tools, primarily due to its user-friendly nature and ability to quantitatively evaluate diverse aspects of environmental sustainability [36]. The ESA score is derived by deducting penalty points from a maximum of 100, based on various factors inherent to the analytical workflow, such as reagent quantities, hazardous properties, energy consumption, and waste generation. Consequently, higher scores, approaching the maximum of 100, are indicative of more environmentally responsible and greener methodologies [37]. In our study, we have calculated the ESA values for the proposed analytical approaches and compared them with those of previously reported methods, as presented in Tables 4 and 5. The results demonstrate that our developed methods exhibit a superior greenness profile, with the highest ESA score of 88 and 92 for UPLC and Spectrophotometric methods respectively. This remarkable score classifies our proposed techniques as excellent green analytical methods, surpassing the environmental sustainability credentials of existing methodologies. While the ESA tool is a valuable and widely used metric for assessing the greenness of analytical methodologies, it is important to recognize its limitations and potential disadvantages including oversimplification, limited scope, subjectivity in scoring, lack of weighting, and static set of criteria and scoring rules, which may not evolve or adapt to emerging sustainability concerns, new technologies, or changing regulatory frameworks. To address these limitations, it is often recommended to complement the ESA tool with other complementary assessment methods.

#### BAGI criteria

Unlike predominantly greenness-focused tools, the recently introduced BAGI metric delivers a quantitative estimation of an analytical method's “blueness”, defined as its real-world fitness for purpose based on critical practical criteria [38]. BAGI facilitates an inclusive evaluation of an analytical process's suitability or "blueness" by considering ten crucial factors: the kind of analysis, the number of analytes used, the instrumentation employed, the sample productivity, the prerequisites for sample preparation, the number of samples examined in an hour, the reagents and materials demanded, the need for pre-concentration steps, the automation level, and the quantity of sample needed. Each of these ten factors is assigned a score ranging from 1 (lowest) to 10 (highest). All ten criteria contribute to the final BAGI score, which is determined by taking the geometric mean of those scores. More relevance, functionality, and fit for the intended purpose are characteristics of an analytical method with a higher BAGI score. In this study, UPLC and spectrophotometric protocols obtained high BAGI scores of 82.5 and 90, respectively, indicating excellent real-world applicability, high throughput, automation potential, and low operation costs. The short analysis times of 1.67 min (FEX) and 3.54 min (MLK) in UPLC and straightforward measurements in derivative spectrophotometry offer significant practical advantages. Moreover, the use of an eco-friendly aqueous micellar mobile phase minimizes material requirements and waste generation, echoing GAC principles within the applied analysis. Hence, the BAGI assessment confirmed the outstanding blueness of the developed methods. However, since BAGI specifically focuses on practical criteria, it does not provide holistic sustainability quantification. Therefore, we additionally employed the RGB12 algorithm to evaluate composite analytical sustainability considering greenness, performance, and practicality. The consistently high scores across tools verify this method's merit as an implementable green chemistry alternative.

#### RGB12 criteria

In the evaluation of the overall sustainability of the proposed methods, termed "whiteness," the RGB12 approach was employed as an effective algorithm for quantitatively assessing the sustainability level of analytical techniques [8]. It is a quantitative assessment metric that is easy to use for evaluating whiteness. This tool determines the degree of sustainability concerning whiteness assessment and assesses methods based on the 12 WAC considerations [39]. Twelve separate algorithms, categorized as "red," "green," and "blue," make up the RGB12 algorithm, where each group consists of four algorithms. The green subgroup (G1-G4) includes essential GAC parameters such as toxicity, amount of waste and reagent, energy conservation, influence on humans and animals, and genetic alterations. The red subgroup (R1-R4) focuses on validation parameters such as application scope, accuracy, LOD, precision, and LOQ. The blue subgroup (B1-B4) concerns practical and economic necessities, cost-effectiveness, and time efficiency. The RGB12 algorithm exemplifies adherence to WAC principles by summing the scores of the method in each of the three-color domains to obtain the ultimate "whiteness" value. As illustrated in Tables 4 and 5, the proposed methods attain remarkable whiteness values of 88.1 and 89.8 for the UPLC and spectrophotometric methods, respectively, in contrast to the lower values observed for the reported methods. Comprehensive RGB12 algorithm evaluation confirms the proposed methodologies' exceptional performance, demonstrating their sustainability and superiority over existing methods, as illustrated in Fig. S16 and Fig. S17. This assessment further highlights the proposed approaches' remarkable performance, dependability, and economic viability, rendering them attractive options for diverse analytical applications. The RGB12 tool's strength is its ability to evaluate an analytical method's alignment with sustainable analytical chemistry principles holistically and multidimensionally, surpassing other assessment methods. RGB12 assesses all relevant sustainability parameters, unlike NEMI, ComplexGAPI, AGREE, ESA, and BAGI, which focus on specific aspects. RGB12 complements these other tools by quantifying how well an analytical approach follows sustainable analytical practices. By using RGB12 and other assessment metrics, this study was able to thoroughly assess the sustainability of the proposed analytical methods. This multipronged approach mitigated the limitations of any single assessment technique, ensuring a more balanced and unbiased evaluation. This research's systems-oriented strategy, which integrated multiple complementary assessment tools, shows how to evaluate analytical method sustainability thoroughly and rigorously without biases or blind spots.

#### Significance of the multi-tool assessment strategy

The study demonstrated the benefits of using multiple complementary tools (NEMI, ComplexGAPI, AGREE, ESA, BAGI, RGB12) to evaluate the sustainability of analytical methods across various dimensions. These tools holistically assessed greenness (environmental impact), blueness (practicality/cost-effectiveness), whiteness (overall sustainability including performance and safety), and other metrics. The developed analytical procedures showed superior sustainability compared to existing methods based on factors like reduced toxicity, waste minimization, material efficiency, high throughput, automation potential, low cost, and excellent analytical performance. The combined qualitative and quantitative evaluation confirmed the new methods as highly sustainable and implementable best practices that align well with the principles of green analytical chemistry across the environmental, economic, and performance domains.

## Conclusion

Driven by sustainable development goals, we successfully developed and validated an eco-friendly micellar UPLC method and complementary spectrophotometric techniques for the pharmaceuticals MLK and FEX. Comprehensive validation demonstrated excellent performance for simultaneous quantification under optimized conditions, with high linearity, accuracy, precision, sensitivity, and selectivity. The successful application of these methods to pharmaceutical samples further proved the suitability of the methods for routine analysis. Additionally, extensive greenness, blueness, and whiteness assessments using tools such as NEMI, ComplexGAPI, AGREE, ESA, BAGI, and RGB12 verified the outstanding sustainability metrics, efficacy, and practical functionality. The use of an eco-friendly aqueous micellar mobile phase reduced solvent usage and waste generation by more than 50% compared to the use of existing methods, echoing green analytical principles. Short run times of less than 3.5 min and operational simplicity offered significant practical advantages, as reflected by high BAGI scores of up to 82.5 and 90 for UPLC and spectrophotometric methods, respectively. Furthermore, the RGB12 sustainability indices up to 88.1 and 89.8 for the UPLC and spectrophotometric methods, respectively, confirmed the excellent composite performance across critical parameters. Therefore, the developed methods align analytical science with sustainable practices by reliably integrating environmental and economic aspirations. This paper reports the successful demonstration of implementable green analytical techniques balancing efficacy and eco-friendliness pioneer pathways for widespread adoption in pharmaceutical quality control. We envision that this work will motivate analytical scientists to proactively embed holistic sustainability considerations when developing new solutions.

### Supplementary Information

Supplementary Material 1.

## Data Availability

The corresponding author will provide the datasets created and/or analyzed during the current study upon reasonable request.
