# Supplementary material for: Green micellar UPLC and complementary eco-friendly spectroscopic techniques for simultaneous analysis of anti-COVID drugs: a comprehensive evaluation of greenness, blueness, and whiteness
Source: BMC Chem. 2024 Aug 9;18(1):149. doi: 10.1186/s13065-024-01254-8 (PMC11312741; doi:10.1186/s13065-024-01254-8)
Supplement: Supplementary file 1 — Supplementary Material 1. [file 13065_2024_1254_MOESM1_ESM.docx]

**Supplementary Tables**

**Table S1.** Optimization of chromatographic conditions for the determination of studied drugs by the proposed micellar UPLC method

| Parameter | | **No of theoretical plates (N)** | | **Tailing factor (T)** | | **Resolution Factor (R_s_)** | |
| --- | --- | --- | --- | --- | --- | --- | --- |
|  |  | MLK | FEX | MLK | FEX | MLK | FEX |
| Conc of SDS | 0.01  0.02  0.03  0.04 | 7214  7260  7271  7201 | 6710  6796  6721  6786 | 1.13  1.19  1.12  1.12 | 1.13  1.14  1.17  1.19 | 4.12  4.27  4.07  4.15 | 4.87  4.92  4.84  4.85 |
| % of pentanol  (% v/v) | 5  10  15  20 | 7258  7168  7260  7111 | 6753  6745  6796  6674 | 1.12  1.09  1.19  1.11 | 1.11  1.15  1.14  1.12 | 4.16  4.24  4.27  4.25 | 4.79  4.82  4.92  4.85 |
| pH | 3  3.5  4  4.5 | 7214  7260  7201  7101 | 6670  6796  6710  6646 | 1.12  1.19  1.11  1.12 | 1.13  1.14  1.13  1.13 | 4.12  4.27  4.17  4.15 | 4.87  4.92  4.90  4.90 |
| Conc of buffer | 0.02  0.03  0.04  0.05 | 7212  7223  7211  7260 | 6710  6766  6770  6796 | 1.13  1.14  1.12  1.19 | 1.11  1.14  1.13  1.14 | 3.97  4.14  4.10  4.27 | 4.87  4.82  4.84  4.92 |
| Flow rate | 0.6  0.8  1.0  1.2 | 7224  7125  7260  7161 | 6710  6576  6796  6636 | 1.13  1.15  1.19  1.12 | 1.13  1.14  1.14  1.08 | 4.12  4.23  4.27  4.15 | 4.68  4.82  4.92  4.55 |
| Column temp. | 30  40  50 | 7124  7260  7151 | 6621  6796  6672 | 1.13  1.19  1.12 | 1.12  1.14  1.13 | 4.07  4.27  3.97 | 4.63  4.92  4.58 |

| **Parameters** | **Obtained value** | | **Reference value** |
| --- | --- | --- | --- |
|  | **MLK** | **FEX** |  |
| Retention time (R_t_) | 3.54 | 1.67 | -- |
| Number of theoretical plates (N) | 7260 | 6796 | N>2000 |
| Resolution factor (R_s_) | 4.92 | | ≥2 |
| Tailing factor (T) | 1.19 | 1.14 | ≤2 |

**Table S2.** System suitability data for the proposed micellar UPLC method

**Table S3: Determination of MLK and FEX in laboratory-prepared mixtures by the proposed methods.**

| **Lab prepared mixture**  **(µg mL^-1^)** | | **Micellar UPLC method** | | **Lab prepared mixture**  **(µg mL^-1^)** | | **Spectroscopic methods** | | | | | | | |
| --- | --- | --- | --- | --- | --- | --- | --- | --- | --- | --- | --- | --- | --- |
|  |  |  |  |  |  | **Second derivative ^2^D** | | **Third derivative ^3^D** | | | | **Ratio Difference RD** | |
| **MLK** | **FEX** | **MLK** | **FEX** | **MLK** | **FEX** | **MLK** | **FEX** | **MLK** | | | **FEX** | **MLK** | **FEX** |
|  |  | **230 nm** | |  |  | **341 nm** | **213.6 nm** | **295 nm** | **336.4 nm** | **346 nm** | **235 nm** | **ΔP 250-245 nm** | **ΔP 227-217 nm** |
| 10 | 10 | 100.32 | 101.21 | 10 | 10 | 101.62 | 100.58 | 98.82 | 99.40 | 101.74 | 99.12 | 101.80 | 100.70 |
| 20 | 20 | 97.85 | 99.54 | 20 | 10 | 101.40 | 99.74 | 102.05 | 100.90 | 98.15 | 98.64 | 101.77 | 100.42 |
| 2 | 14 | 102.12 | 99.76 | 10 | 20 | 98.15 | 98.45 | 100.76 | 99.61 | 101.33 | 101.56 | 98.78 | 99.52 |
| 5 | 60 | 98.26 | 100.65 | 3 | 36 | 101.30 | 99.41 | 98.93 | 100.09 | 99.83 | 100.33 | 101.42 | 100.30 |
| 20 | 240 | 98.34 | 100.87 | 4 | 48 | 98.72 | 100.64 | 101.01 | 101.00 | 101.08 | 99.9 | 102.04 | 99.63 |
|  |  |  |  | 5 | 60 | 98.65 | 101.39 | 100.48 | 99.34 | 100.72 | 101.87 | 100.08 | 98.85 |
| **Mean %**  **± SD** | | 98.42  ±1.68 | 100.40  **±**722 | **Mean %**  **± SD** | | 99.97  ±1.62 | 100.04  ±1.05 | 100.34  ±1.25 | 100.06  ±0.74 | 100.48  ±1.31 | 100.24  ±1.29 | 100.98  ±1.29 | 99.90  ± 0.69 |

-Reported method (Mustafa et al., 2017) for determination of Montelukast sodium nm

**Supplementary Figure**

**Fig. S1:** (A) Ratio spectra of MLK 3- 50 µg mL^-1^ using 15 µg mL^-1^ FEX as divisor. (B) Ratio spectra of FEX 3- 60 µg mL^-1^ using 5 µg mL^-1^ MLK as divisor


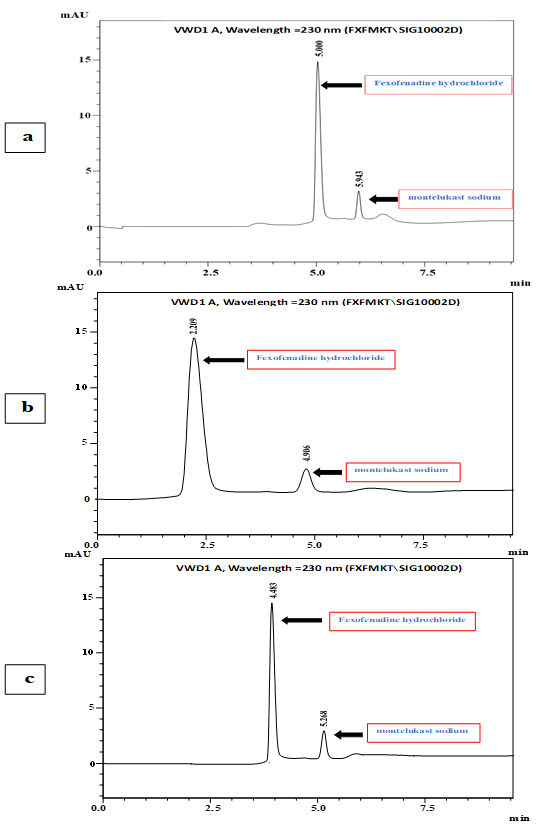


**Fig. S2:** Optimization chromatograms of selection of column demonstrating the separation of 4 μg mL^-1^ MLK and 48 μg mL^-1^ FEX laboratory-prepared mixture. (a) Kinetex C18 (100 x 4.6 mm, 2.6 μm), (b) Zorbax Eclipse XDB-C18 (150 x 4.6 mm, 5 μm), and (c) Monolithic RP-C18 (100 x 4.6 mm).


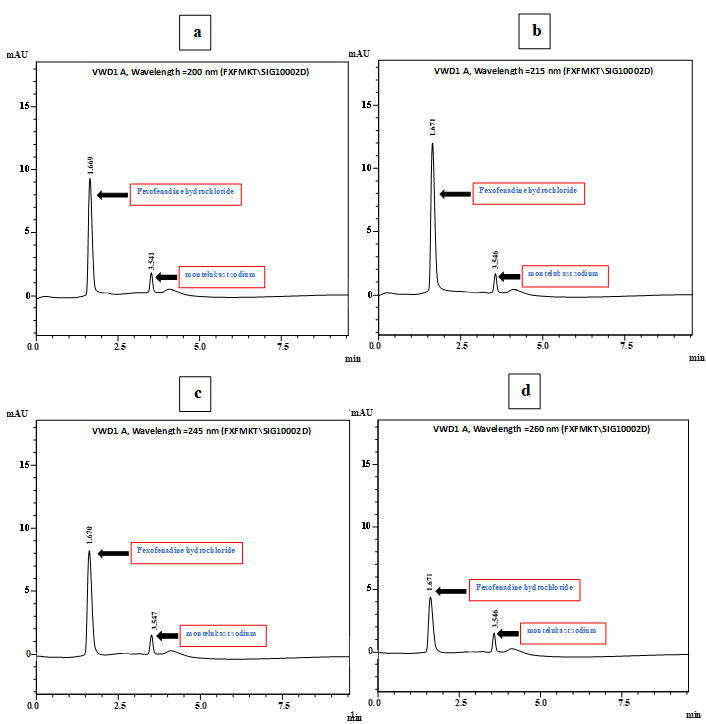


**Fig. S3:** Optimization chromatograms of selection of detection wavelength demonstrating the separation of 4 μg mL^-1^ MLK and 48 μg mL^-1^ FEX laboratory-prepared mixture. (a) 200 nm, (b) 215 nm, (c) 245 nm, and (d) 260 nm.


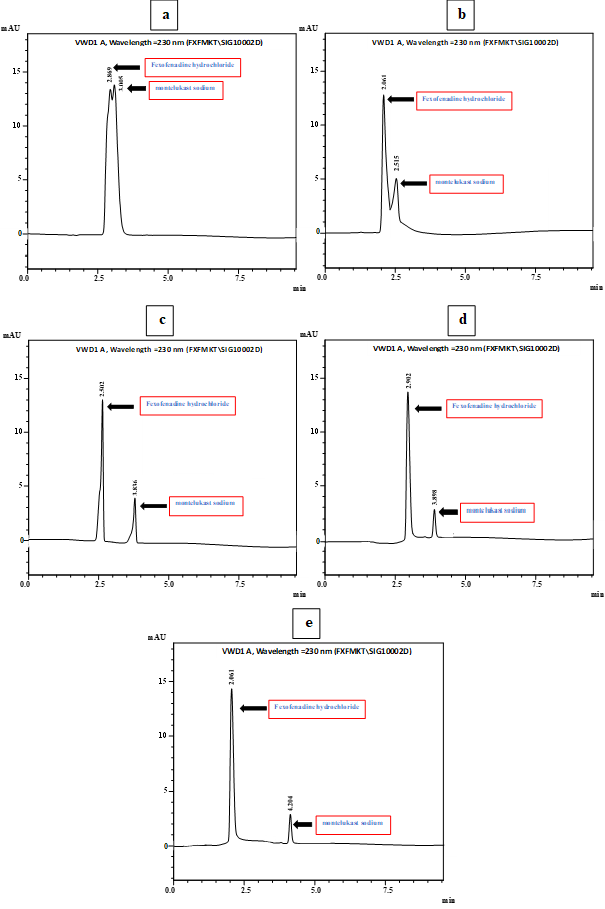


**Fig. S4:** Optimization chromatograms of selection of mobile phase organic modifiers demonstrating the separation of 4 μg mL^-1^ MLK and 48 μg mL^-1^ FEX laboratory-prepared mixture. (a) methanol, (b) ethanol, (c) acetonitrile, (d) n-propanol, and (e) n-butanol


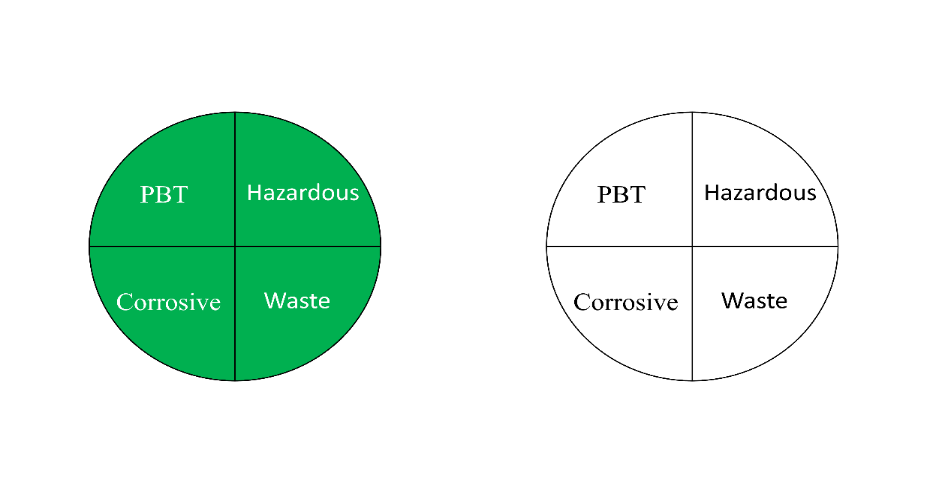


**Fig. S5.** Typical NEMI pictograms.

.


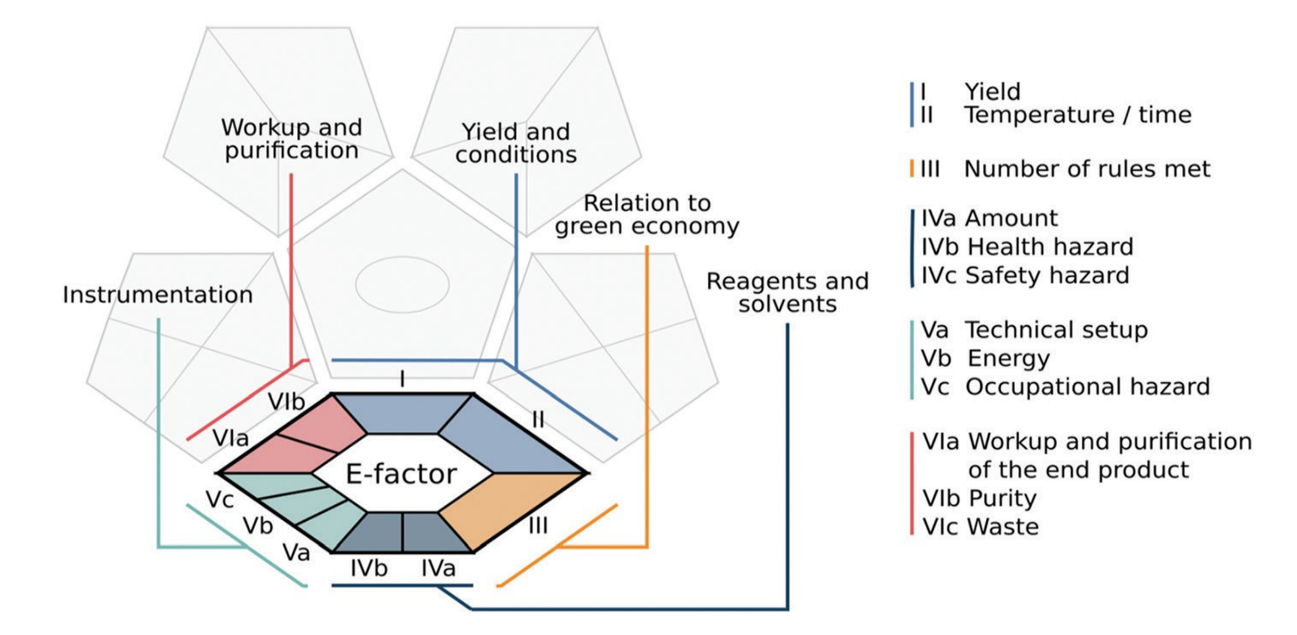


**Fig. S6.** The ComplexGAPI pictogram, with the original GAPI pictogram greyed out in the background, and particular fields of the added hexagonal glyph grouped and colour-coded for clarity.

**
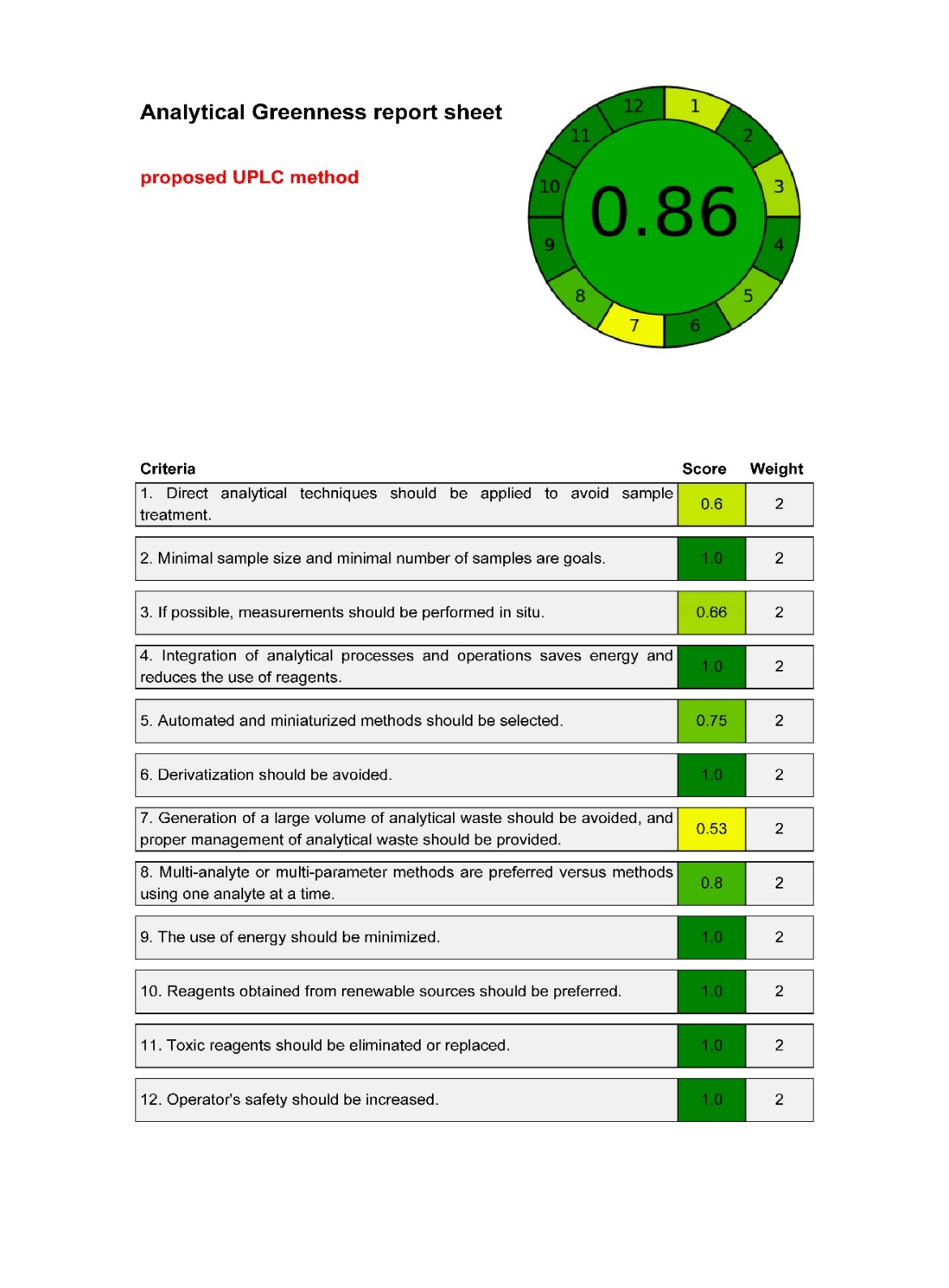
**

**Fig. S7.** Analytical greenness report sheets for the **proposed UPLC** **method** by AGREE tool.


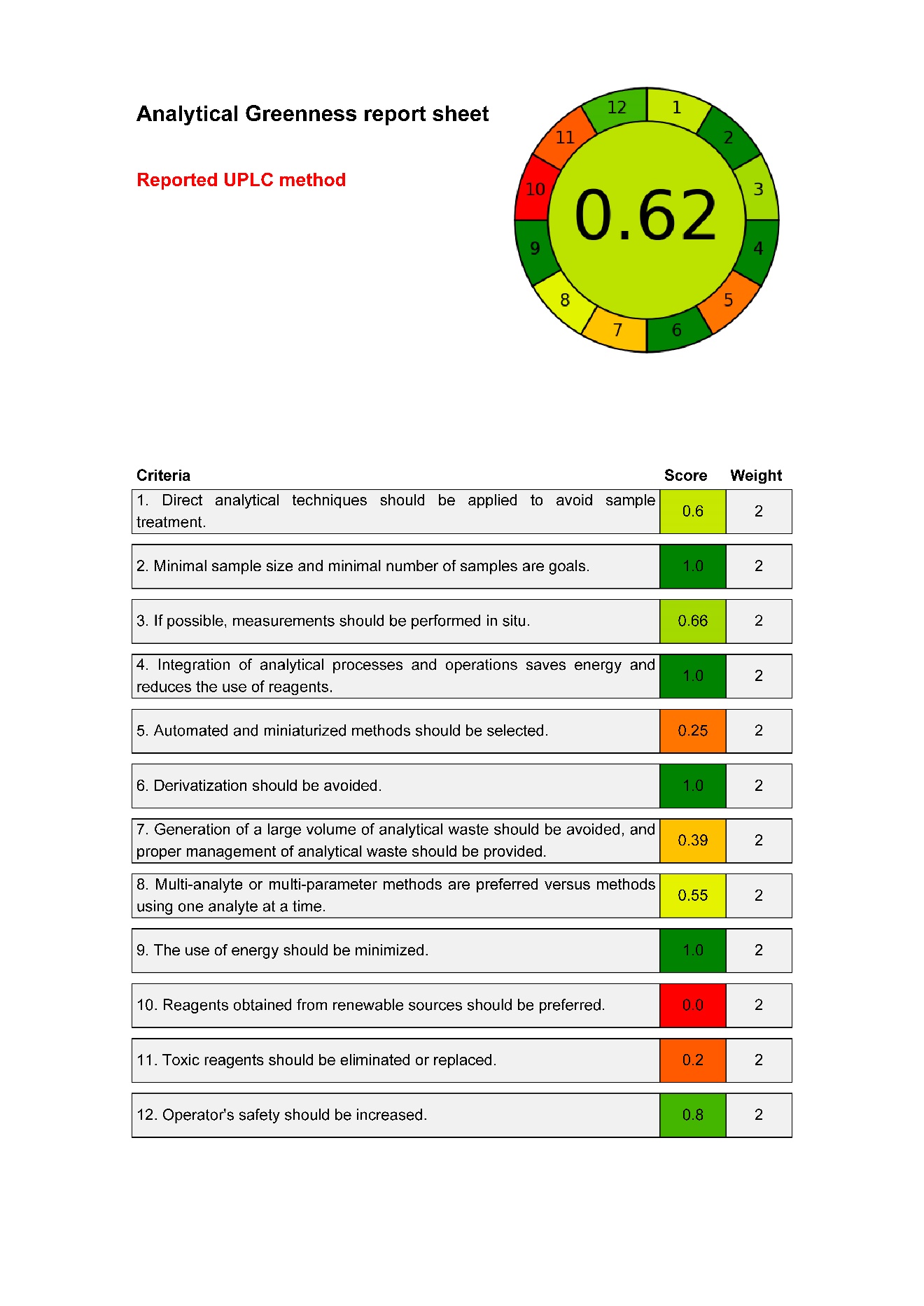


**Fig. S8.** Analytical greenness report sheets for the **reported UPLC method [25]** by AGREE tool.


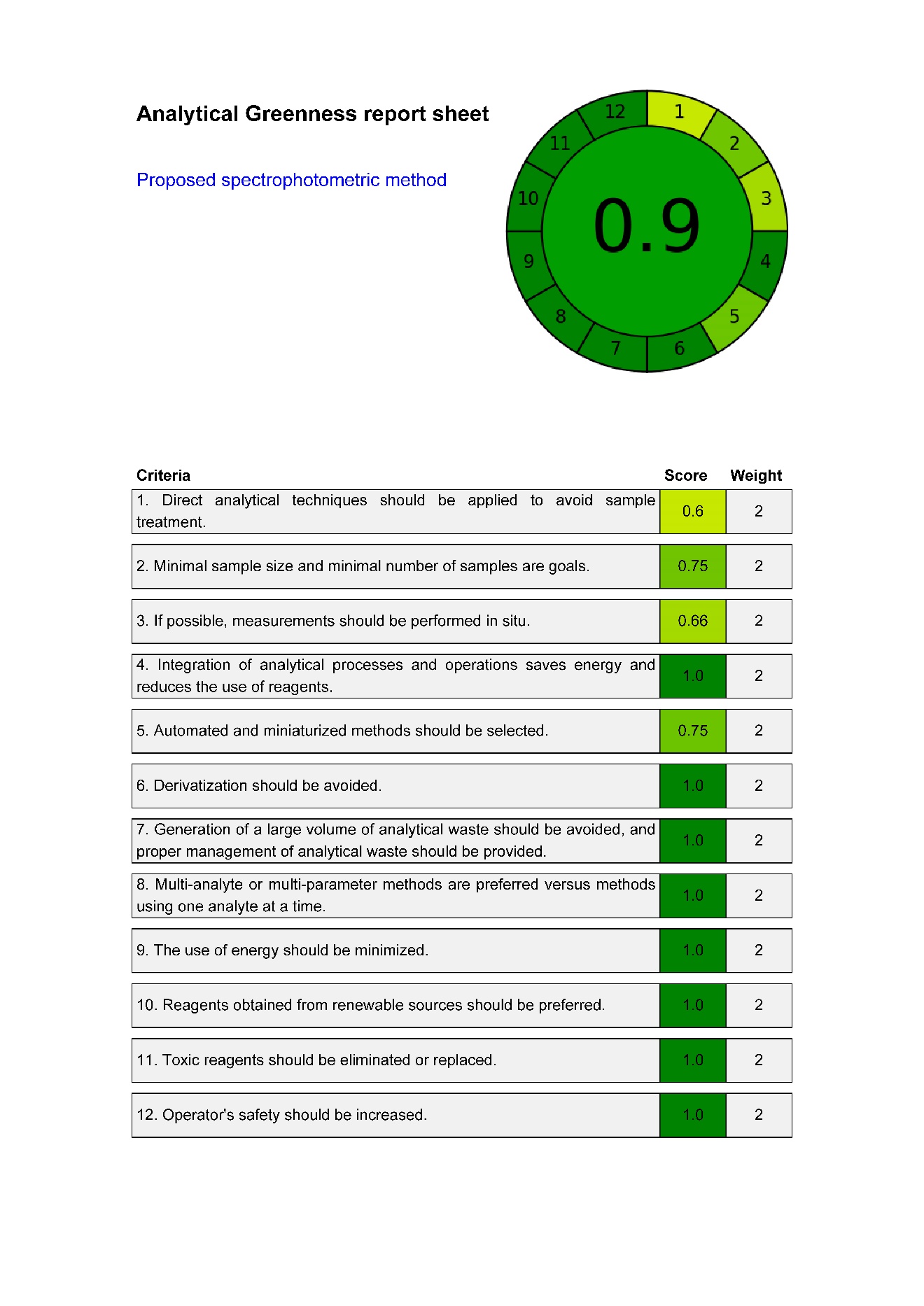


**Fig. S9.** Analytical greenness report sheets for the **proposed spectrophotometric** **method** by AGREE tool.


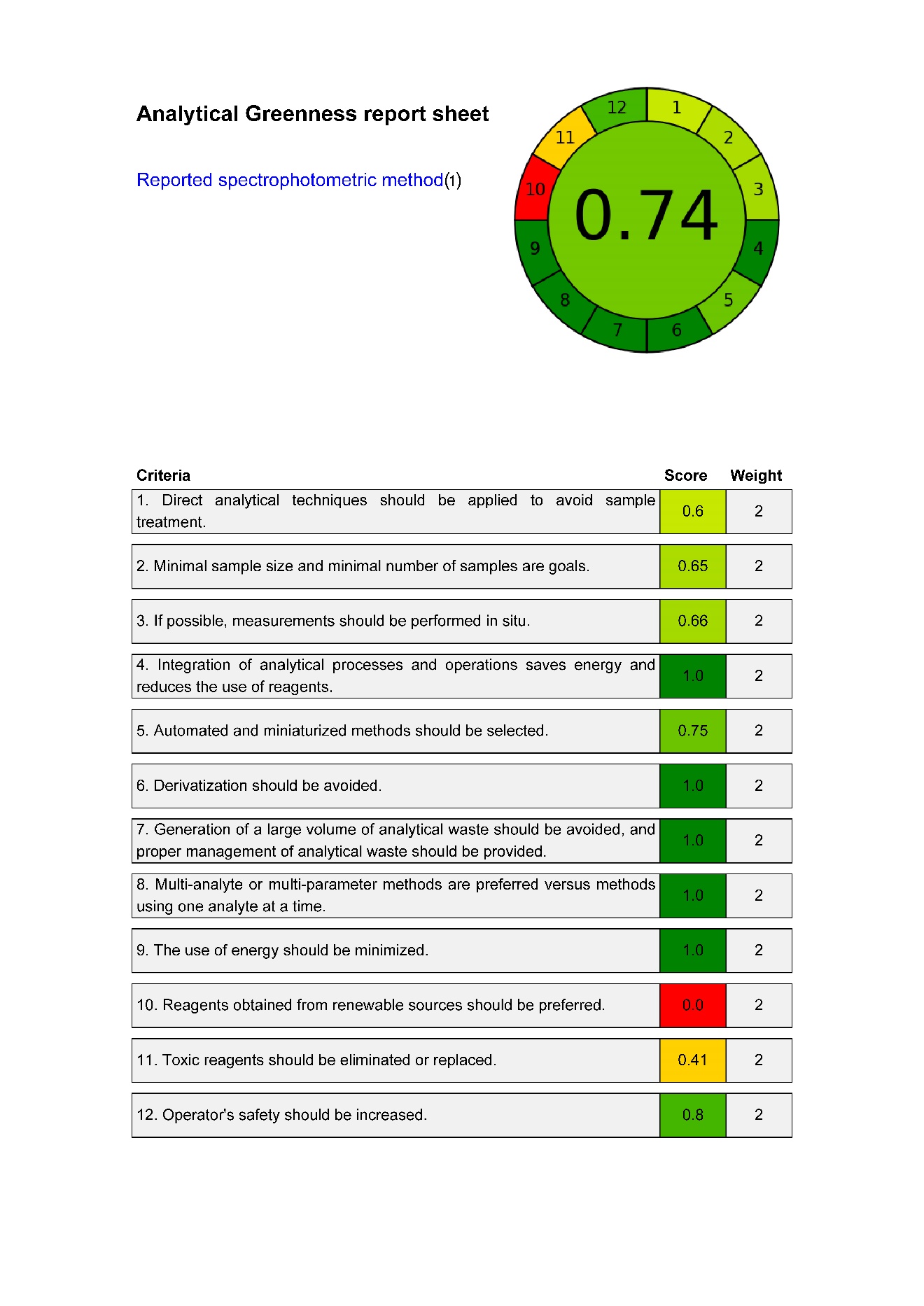


**Fig. S10.** Analytical greenness report sheets for the **reported spectrophotometric** **method** **[15]** by AGREE tool.


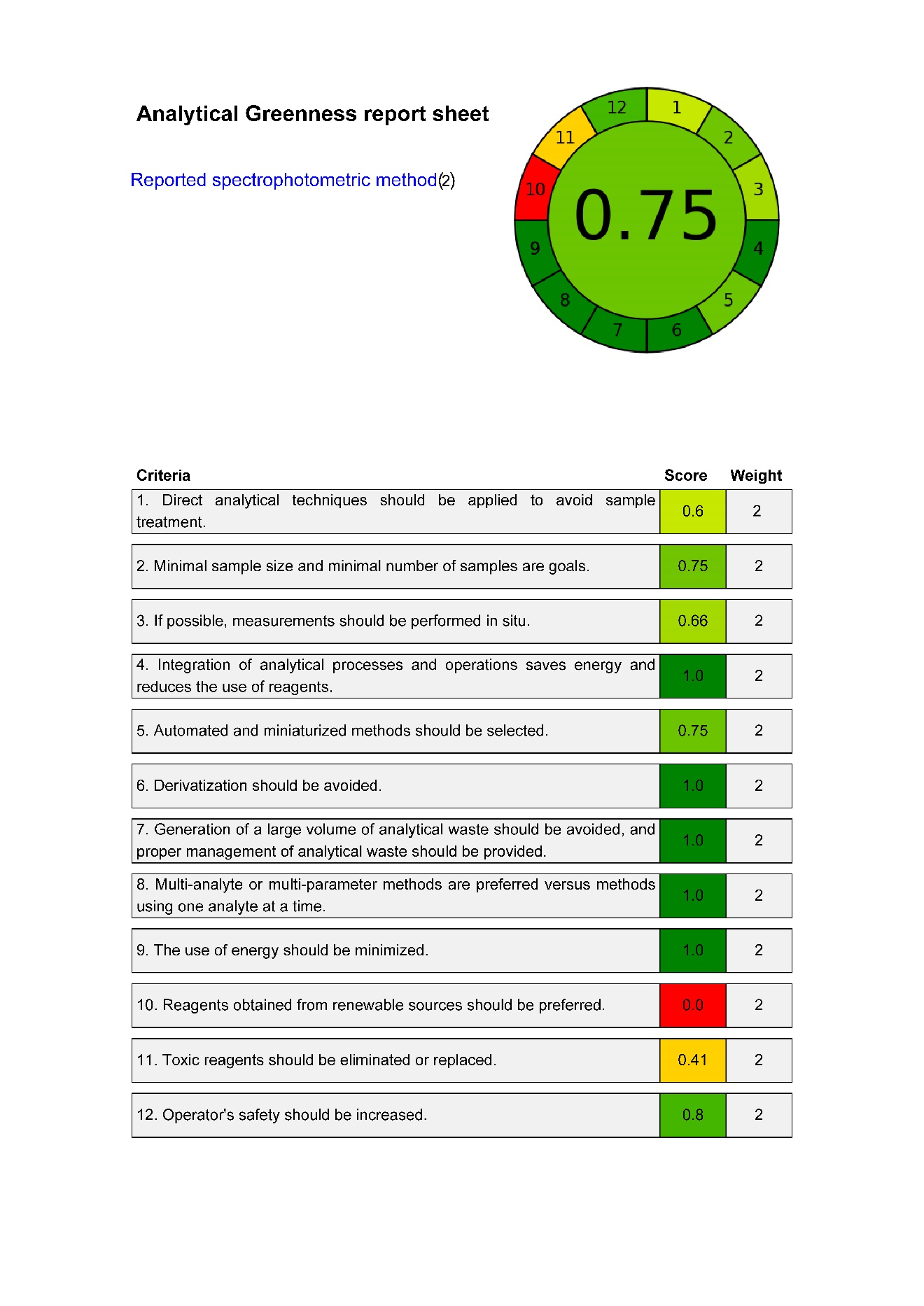


**Fig. S11.** Analytical greenness report sheets for the **reported spectrophotometric** **method** **[16]** by AGREE tool.


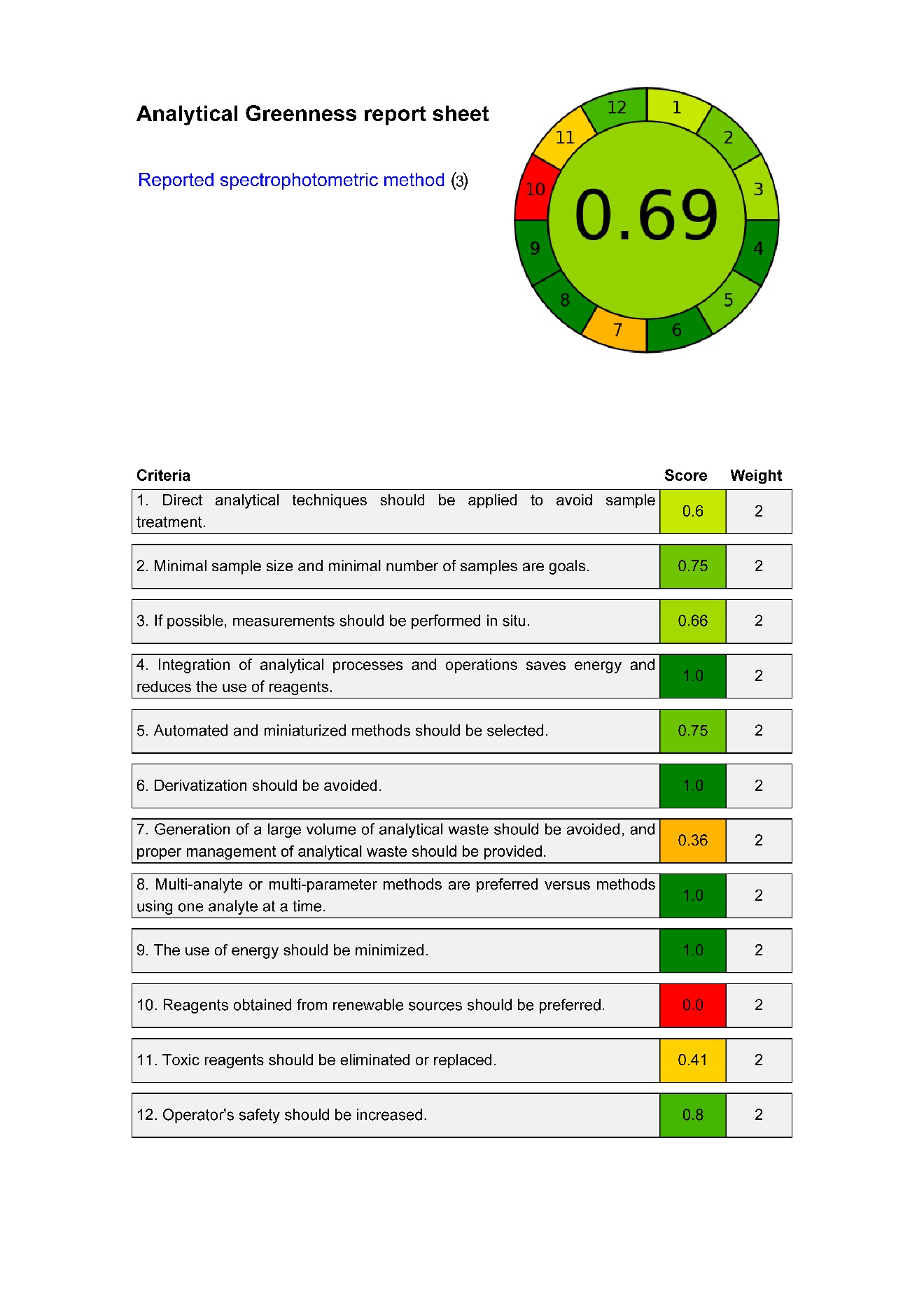


**Fig. S12.** Analytical greenness report sheets for the **reported spectrophotometric** **method** **[17]** by AGREE tool.


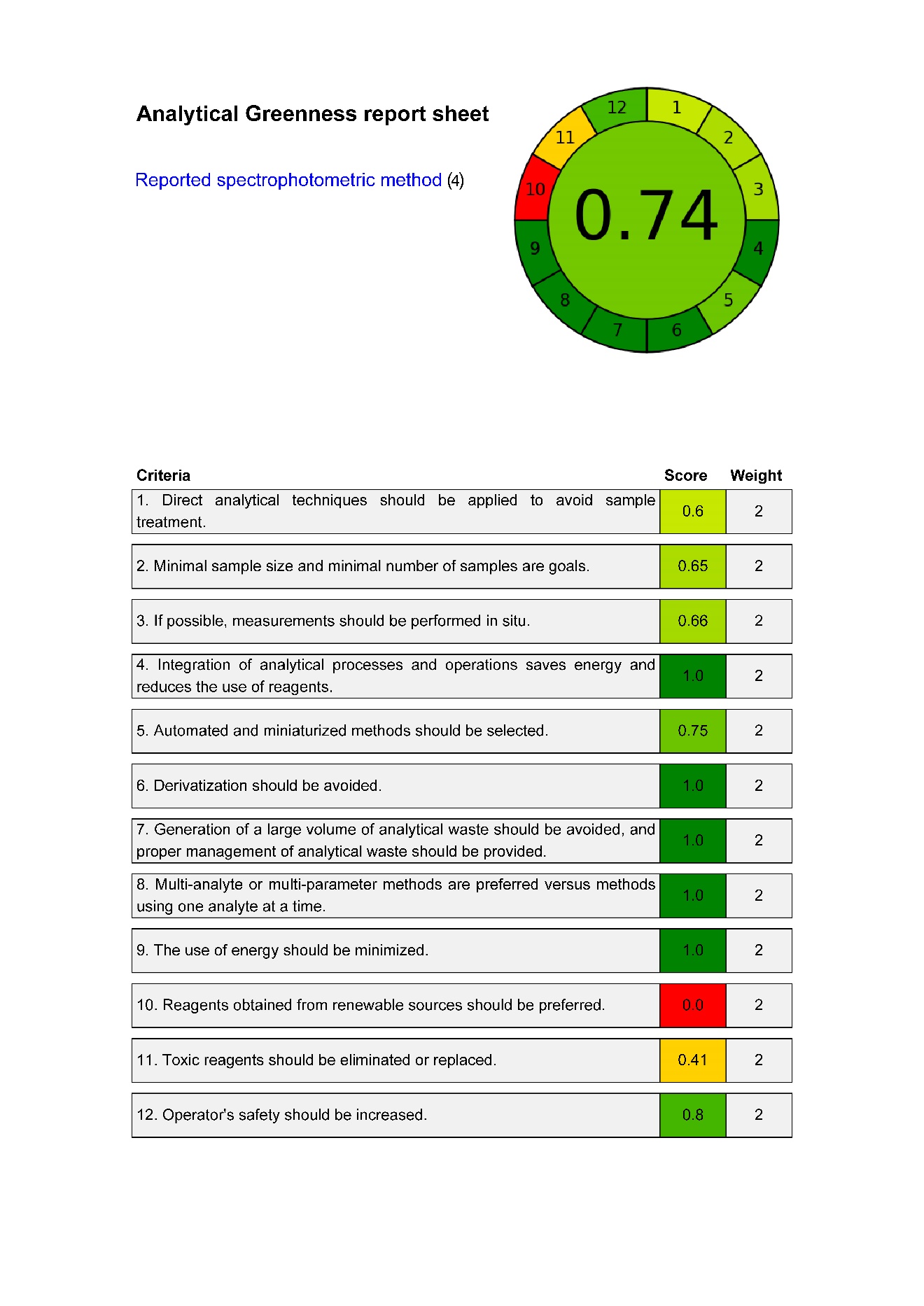


**Fig. S13.** Analytical greenness report sheets for the **reported spectrophotometric** **method** **[18]** by AGREE tool.


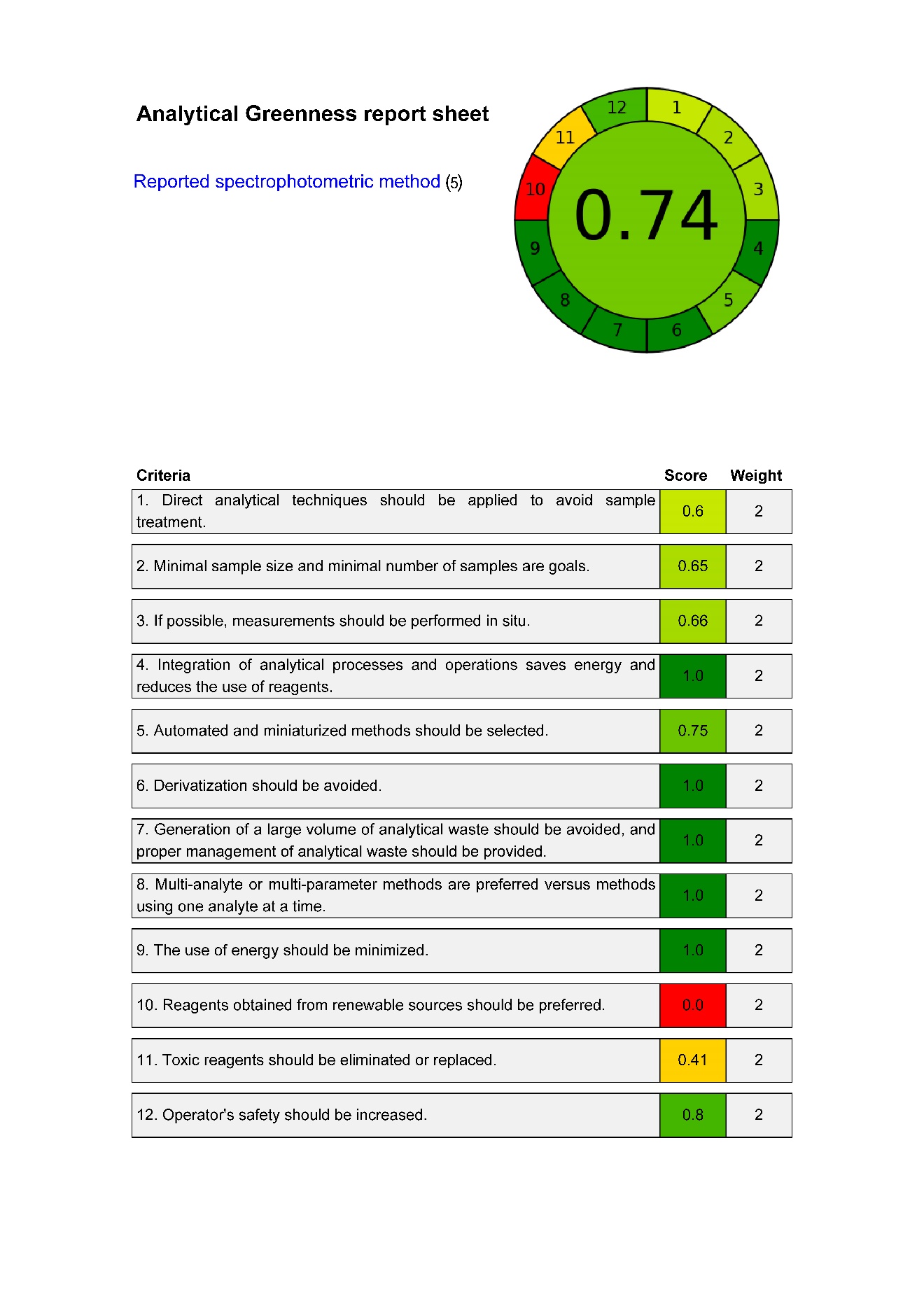


**Fig. S14.** Analytical greenness report sheets for the **reported spectrophotometric** **method** **[19]** by AGREE tool.


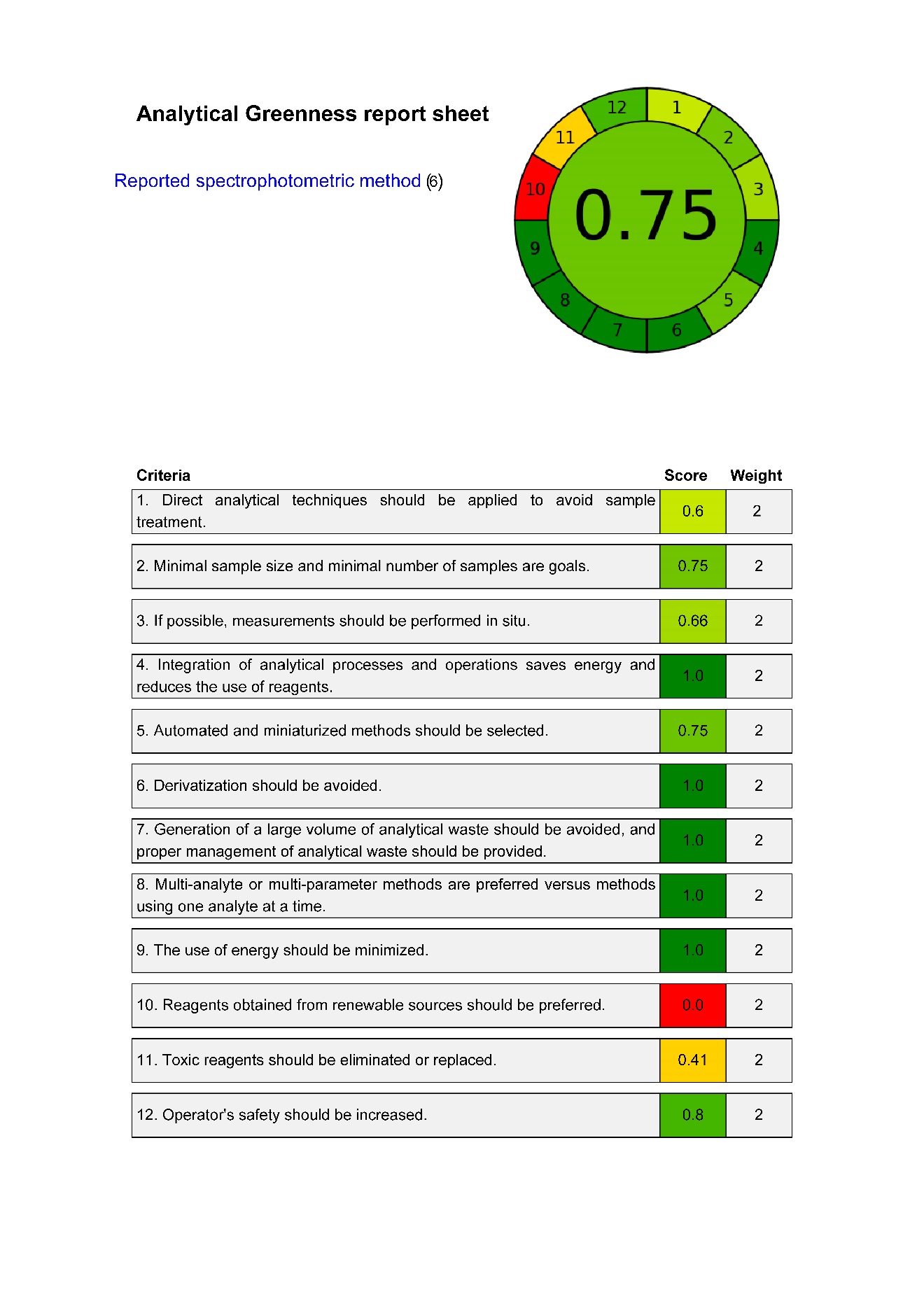


**Fig. S15.** Analytical greenness report sheets for the **reported spectrophotometric** **method** **[20]** by AGREE tool.


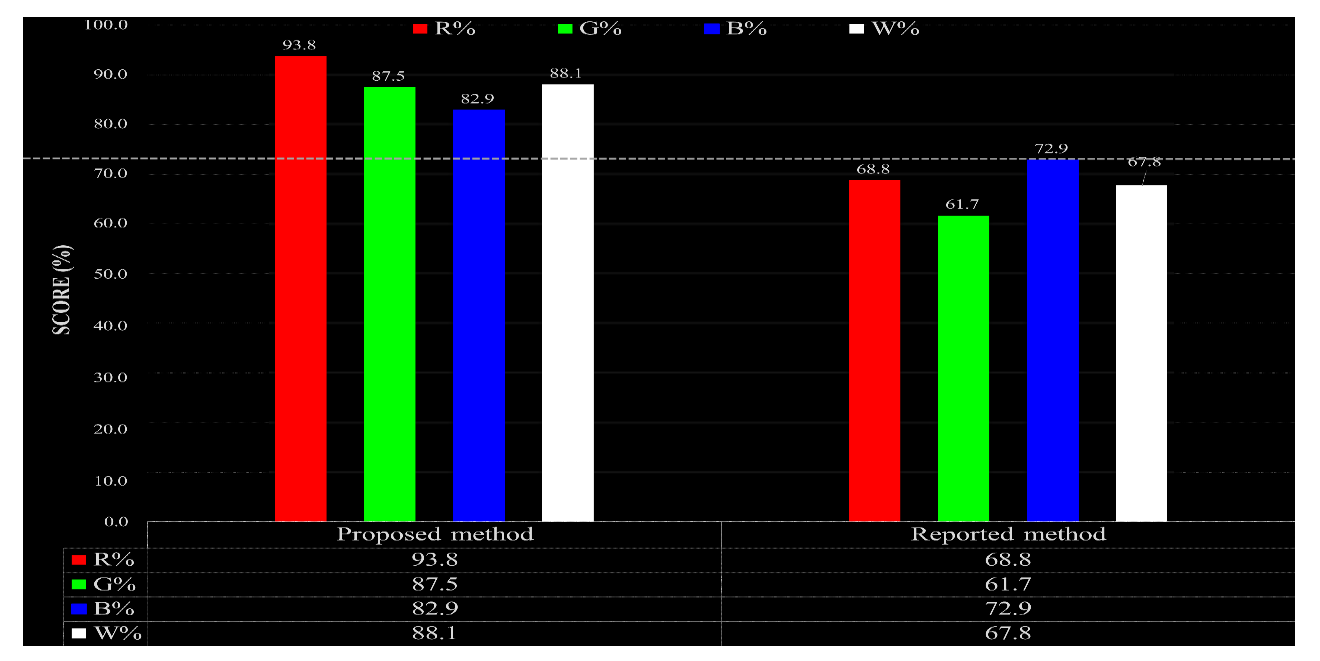


**Fig. S16.** Comparison of the redness, greenness, blueness, and whiteness profiles of the proposed and reported UPLC methods, obtained by the RGB 12 algorithm.


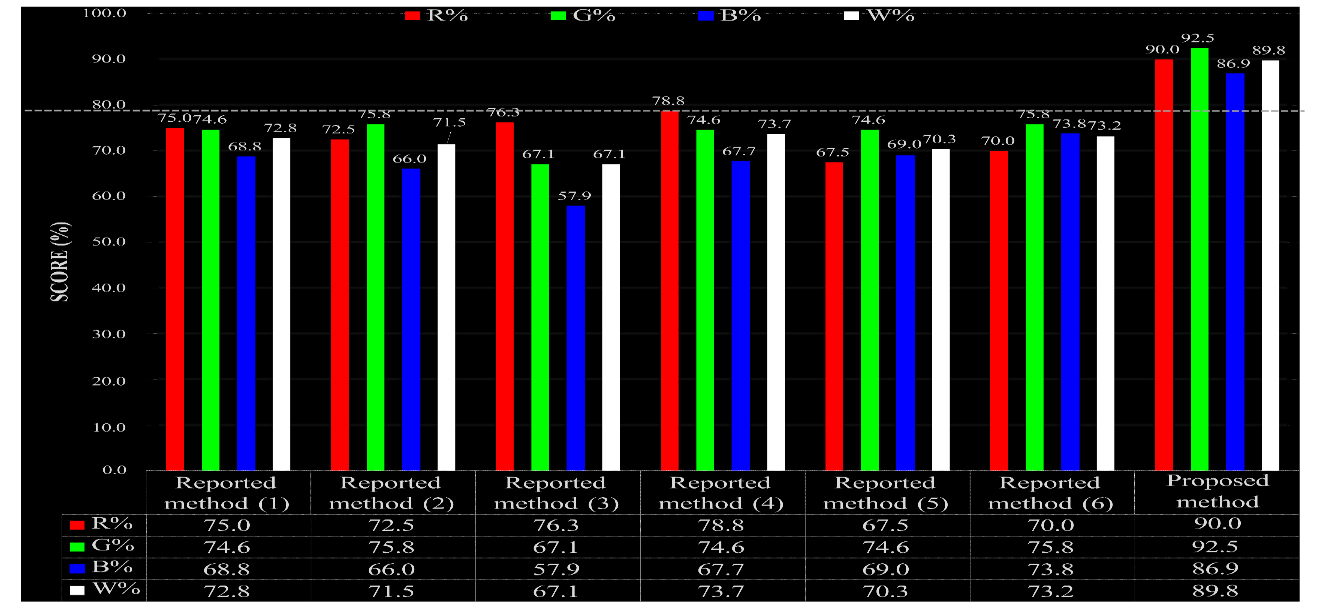


**Fig. S17.** Comparison of the redness, greenness, blueness, and whiteness profiles of the proposed and reported spectrophotometric methods, obtained by the RGB 12 algorithm.
